# Whole-genome sequencing analysis of two heat-evolved *Escherichia coli* strains

**DOI:** 10.1186/s12864-023-09266-9

**Published:** 2023-03-27

**Authors:** Bailey E. McGuire, Francis E. Nano

**Affiliations:** Department of Biochemistry and Microbiology, University of Victoria, Victoria, B.C Canada

**Keywords:** Chaperone, Directed evolution, Adaptive laboratory evolution, Genomics, Thermotolerance, Chromosomal rearrangement

## Abstract

**Background:**

High temperatures cause a suite of problems for cells, including protein unfolding and aggregation; increased membrane fluidity; and changes in DNA supercoiling, RNA stability, transcription and translation. Consequently, enhanced thermotolerance can evolve through an unknown number of genetic mechanisms even in the simple model bacterium *Escherichia coli*. To date, each *E. coli* study exploring this question resulted in a different set of mutations. To understand the changes that can arise when an organism evolves to grow at higher temperatures, we sequenced and analyzed two previously described *E. coli* strains, BM28 and BM28 *ΔlysU*, that have been laboratory adapted to the highest *E. coli* growth temperature reported to date.

**Results:**

We found three large deletions in the BM28 and BM28 *ΔlysU* strains of 123, 15 and 8.5 kb in length and an expansion of IS10 elements. We found that BM28 and BM28 *ΔlysU* have considerably different genomes, suggesting that the BM28 culture that gave rise to BM28 and BM28 Δ*lysU* was a mixed population of genetically different cells. Consistent with published findings of high GroESL expression in BM28, we found that BM28 inexplicitly carries the *groESL* bearing plasmid pOF39 that was maintained simply by high-temperature selection pressure. We identified over 200 smaller insertions, deletions, single nucleotide polymorphisms and other mutations, including changes in master regulators such as the RNA polymerase and the transcriptional termination factor Rho. Importantly, this genome analysis demonstrates that the commonly cited findings that LysU plays a crucial role in thermotolerance and that GroESL hyper-expression is brought about by chromosomal mutations are based on a previous misinterpretation of the genotype of BM28.

**Conclusions:**

This whole-genome sequencing study describes genetically distinct mechanisms of thermotolerance evolution from those found in other heat-evolved *E. coli* strains. Studying adaptive laboratory evolution to heat in simple model organisms is important in the context of climate change. It is important to better understand genetic mechanisms of enhancing thermotolerance in bacteria and other organisms, both in terms of optimizing laboratory evolution methods for various organisms and in terms of potential genetic engineering of organisms most at risk or most important to our societies and ecosystems.

**Supplementary Information:**

The online version contains supplementary material available at 10.1186/s12864-023-09266-9.

## Background

With climate change many organisms will have to evolve to survive increasingly warmer environmental niches. It is important for us to understand the nature of these adaptations so that we can monitor ecosystem health, and, in some cases, such as food crops, possibly intervene to accelerate adaptation. Temperature affects the functioning and integrity of important biomolecules in a cell, including DNA, RNA, proteins and lipids. Thus, temperatures around the minimum or maximum growth temperatures (T_min_ or T_max_) of an organism present many problems, requiring the induction of cold shock or heat shock responses for organism survival. High temperatures can unfold or misfold proteins, mRNAs and structural RNAs; lead to aggregation of proteins due to unfolding or misfolding; cause increased membrane fluidity and permeability and cause changes in DNA topology and genome structure. To compensate for these effects, heat-loving organisms, or thermophiles, have more stable proteins and structural RNAs [1, 2], different membrane compositions [3] (e.g., more saturated, long chain and branched chain fatty acids [4]) and other variations compared to moderate-temperature-loving mesophiles or cold-loving psychrophiles. Thermophilic proteins can have enhanced core packing [5], more charged and hydrophobic residues and less polar residues [5–7], and more residues involved in secondary structure elements [7] and other interactions such as salt bridges [5, 7, 8].

Several *Escherichia coli* studies [9–13] have pushed the cells towards higher growth temperatures, successfully increasing the natural T_max_ of ~45.5 °C in rich liquid medium, ~46.5 °C on rich solid medium or ~43–44 °C in liquid media without methionine by up to 3 °C. These impressive feats were accomplished by adaptive laboratory evolution (ALE) to heat, where the cells were grown at progressively higher temperatures, often with simultaneous hyper-mutagenesis. In our interpretation of the literature, the earliest of these studies resulted in the highest T_max_ reported to date [9]. In the study, Rudolph et al. sequenced select heat shock genes and their promoter regions but whole-genome sequencing (WGS) of the heat-evolved strain was not performed. In the 2012 study by Blaby et al. [10], they found a deletion of the glycerol transporter *glpF* and showed that deletion of *glpF* significantly enhanced thermotolerance in the MG1655 wildtype background, and they found that a *fabA* fatty acid desaturase/isomerase mutation increased the amount of saturated and decreased the amount of unsaturated fatty acids in the membrane. However, other mutations in the strain are probably important for the enhanced thermotolerance, including perhaps the mutations in the bifunctional (p)ppGpp synthase/hydrolase *spoT* and the housekeeping σ factor *rpoD*. In a study by Luan et al. published in 2015 [11], the authors found hundreds of changes compared to the DH5α starting strain, including mutations in the alternate σ factor *rpoS* and the transcriptional repressor *cytR*. For the two remaining studies [12, 13], researchers found mutations in *spoT* for all strains or populations, and changes in the RNA polymerase beta’ subunit *rpoC*, *rpoD,* the transcriptional terminator *rho* and in or around the chaperone *groESL* in some strains. In less related studies in minimal media, *E. coli* strains were adapted to constant temperatures between the optimum growth temperature (T_opt_, 37 °C) and the T_max_ (~43–44 °C) growth temperature, at 42 or 42.2 °C [14–18]. In these studies, major parts of the transcriptional machinery are often found mutated, usually either the RNA polymerase beta subunit *rpoB* or *rho*, but not both [14–17]. Amazingly, single amino acid substitutions in these master regulators lead to changes in gene expression for thousands of genes [16, 17].

Altogether, these studies demonstrate that adaptation to heat occurs through many different mechanisms dependent on the starting genomic background. And while all of these researchers were interested in adapting *E. coli* to heat, it is important to note that these strains were likely also adapting to the media composition, usually growth in liquid media rather than on solid media, the growth phase(s) the cells found themselves in, etc. Some studies attempted to control for these other factors by evolving another strain derived from the same parent alongside, under the same conditions except at 37 °C, or by evolving the cells in minimal media to avoid any metabolism-related mutations that may occur in rich media such as LB but that are not associated with heat adaptation. Importantly, *E. coli*’s T_max_ decreases by ~2 °C in media which does not contain methionine, such as many minimal media formulations. This is because the homoserine *O*-succinyltransferase MetA, the first enzyme in the methionine biosynthesis pathway, begins to unfold at temperatures as low as 25 °C [19]. Previous studies [20, 21] have shown that *metA* mutagenesis or replacement of *metA* with a thermophilic homolog increases the thermotolerance of *E. coli* in minimal media. These studies hint at immense differences between adapting *E. coli* to heat in minimal versus rich media, both in terms of growth parameters such as the T_max_ and in terms of which genes can be inactivated while still maintaining growth.

In this paper, we obtain, process and analyze WGS data from the heat-evolved isolates BM28 and BM28 *ΔlysU* from the Rudolph et al. study [9]. These strains have the highest reported liquid T_max_ of the few studies of their kind, and they are the only strains from such studies that have not been analyzed by WGS, so we are very interested in studying their genomes. We discovered that BM28 carry a *groESL* plasmid known as pOF39, which alone increases the T_max_ of MG1655. We describe large chromosomal deletions, an insertion sequence expansion event and over 200 smaller indels and single nucleotide polymorphisms (SNPs) in the strains. To our surprise, we found that BM28 and BM28 *ΔlysU* are considerably different, and we suspect that BM28 cultures were a mix of genetically different cells evolving alongside each other. We identified changes in and around essential, master regulator, heat shock and other stress response genes. Finally, we discuss potential mechanisms for some of the changes that arose and highlight specific changes that may be important to BM28’s and BM28 *ΔlysU*’s thermotolerant phenotypes. Importantly, this study clarifies a few results the original paper is commonly cited for: the overexpression of GroESL is due to pOF39 and not chromosomal mutations acquired through adaptive laboratory evolution to heat, and LysU is likely not critical to thermotolerance.

## Results

### Building the BM28 and BM28 *ΔlysU* genomes

We obtained Illumina short read and Oxford Nanopore long read WGS data from the Microbial Genome Sequencing Center in Pittsburgh, USA. After trimming, the short read data was aligned to the MG1655 reference genome (NC_000913), resulting in a mean coverage of 133.9 per base for BM28 and 85.0 for BM28 *ΔlysU* (see Supplementary Table S1 for more Illumina data statistics). We obtained long read WGS data for BM28 only, resulting in a mean coverage of 587.2 per base, a mean read length of 2.9 kb and maximum read length of 98.5 kb (see Supplementary Table S2 for more Oxford Nanopore data statistics). Using the SPAdes algorithm and the short and long read BM28 data, we were able to generate a single linear de novo assembly scaffold encompassing the whole BM28 genome. This linear scaffold had 613 bp of identical sequence on each end, so we circularized the sequence and deleted one of the 613 bp repeats (located between genes *eco* and *mqo*) to form the BM28 genome (Genbank CP102380.1). Using the BM28 genome as a starting point, we built a genome for BM28 *ΔlysU* (Genbank CP102379.1).

### The parent strain of BM28 and BM28 *ΔlysU* was JB41

Rudolph et al. reported the parent strain of BM28 and BM28 *ΔlysU* as MG1655 *zba::kan* from a Bardwell and Craig paper published in 1988 [22]. Many MG1655-based strains with a *kan* insertion in that position (within *mscK*) were generated in that study, though none were simply MG1655 *zba::kan*. Based on the BM28 and BM28 *ΔlysU* genomes, it appears that the parent strain was JB41, with the reported genotype MG1655 Δ(*gpt-proAB-arg-lac*)XIII *zaj*::Tn10 *zba-315::kan*. However, the Δ(*gpt-proAB-arg-lac*)XIII deletion is not a single genetic element and is probably an artifact of genotyping cells before WGS was available. It seems that Δ(*gpt-proAB-arg-lac*)XIII is really composed of three separate genetic changes: Δ(*gpt-proA*), *lacY1* or *lacZ4*, and *argE3*(Oc), which are all far enough away from each other on the chromosome that it is likely that only one would be transferred via P1 transduction. Thus, the JB41 genotype was likely MG1655 *lacY1* or *lacZ4 zaj::*Tn10 *zba-315::kan* (or *mscK::kan*). We built a reconstruction of the JB41 genome with the *lacY1* mutation and a wildtype Tn10 sequence and submitted it to Genbank (Genbank CP102378.1).

### BM28 and BM28 *ΔlysU* have three large chromosomal deletions

**Fig. 1:**
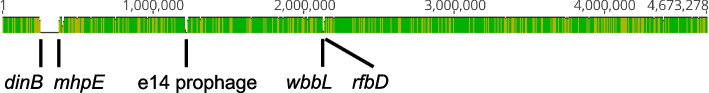
Mauve genome alignment [23] of the MG1655, BM28 and BM28 *ΔlysU* genomes showing the level of identity. The numbering corresponds to the MG1655 genome. There are three gaps in identity that correspond to three large deletions in BM28 and BM28 *ΔlysU*, of lengths 123 kb (from *dinB* to *mhpE*), 15 kb (the e14 prophage) and 8.5 kb (from *wbbL* to *rfbD*)

We aligned the MG1655, BM28 and BM28 *ΔlysU* genomes with Mauve [23] and visualized the level of identity (Fig. 1). Compared to MG1655, BM28 and BM28 *ΔlysU* have three large deletions approximately 123 kb, 15 kb and 8.5 kb in length (Fig. 1 and Supplementary Table S3). The 123 kb deletion spans MG1655 genomic positions 0.251–0.374 Mb, beginning within the *dinB* gene, ending within the *mhpE* gene, and fully deleting the 132 genes between them. This deletion creates a gene encoding a DinB-MhpE fusion protein, including amino acids 1–152 of DinB and residues 80–337 of MhpE. Interestingly, residues 149–152 of DinB and 80–83 of MhpE are identical (AKIA) and the two genes share 12 bp of identical DNA sequence in this region (Supplementary Fig. S1). Thus, we inferred that this deletion resulted from homologous recombination between these 12 bp of identical sequence, deleting 123 kb in the process.

The 15 kb deletion represents a precise deletion of the cryptic prophage e14 which contains 24 genes (Fig. 1 and Supplementary Table S3). This prophage exists at 1.197–1.211 Mb in MG1655, is flanked by host genes *icd* and *icdC* and is excised upon induction of the SOS (DNA damage) response. In a study demonstrating that cryptic prophages contribute to resistance to various stressors [24], Wang et al. showed that in the K-12 strain BW25113, deletion of the e14 prophage did not reduce the cells’ viability to heat shock (at 65 °C for 10 min). As BM28 is a K-12 (MG1655) derivative, the e14 prophage may also not be important for high temperature growth and/or survival in BM28 strains nor their ancestors. The other large deletions and insertion sequence transposition events discussed later likely caused the excision of the e14 prophage, through triggering the SOS response.

Finally, the 8.5 kb deletion from 2.103–2.111 Mb in MG1655 occurs within a cluster of O-antigen synthesis genes, deleting eight genes and partially deleting the IS5-interrupted *wbbL* and uninterrupted *rfbD* genes (Fig. 1 and Supplementary Table S3). Due to the interruption of *wbbL* in MG1655, BM28 and BM28 *ΔlysU* do not produce an O-antigen and instead only produce the lipid A and core regions of lipopolysaccharide [25]. The exact site of the 8.5 kb deletion and reclosing of the chromosome is between the first base of an insertion sequence element IS5 and a base of *rfbD* (Supplementary Fig. S2). Thus, it is possible that an additional IS5 element in the same orientation as the one in *wbbL* was inserted into *rfbD*, and then the 8.5 kb of DNA was deleted through homologous recombination between the two IS5 elements.

### BM28 and BM28 *ΔlysU* likely evolved separately for the last year of the experiment

**Fig. 2:**
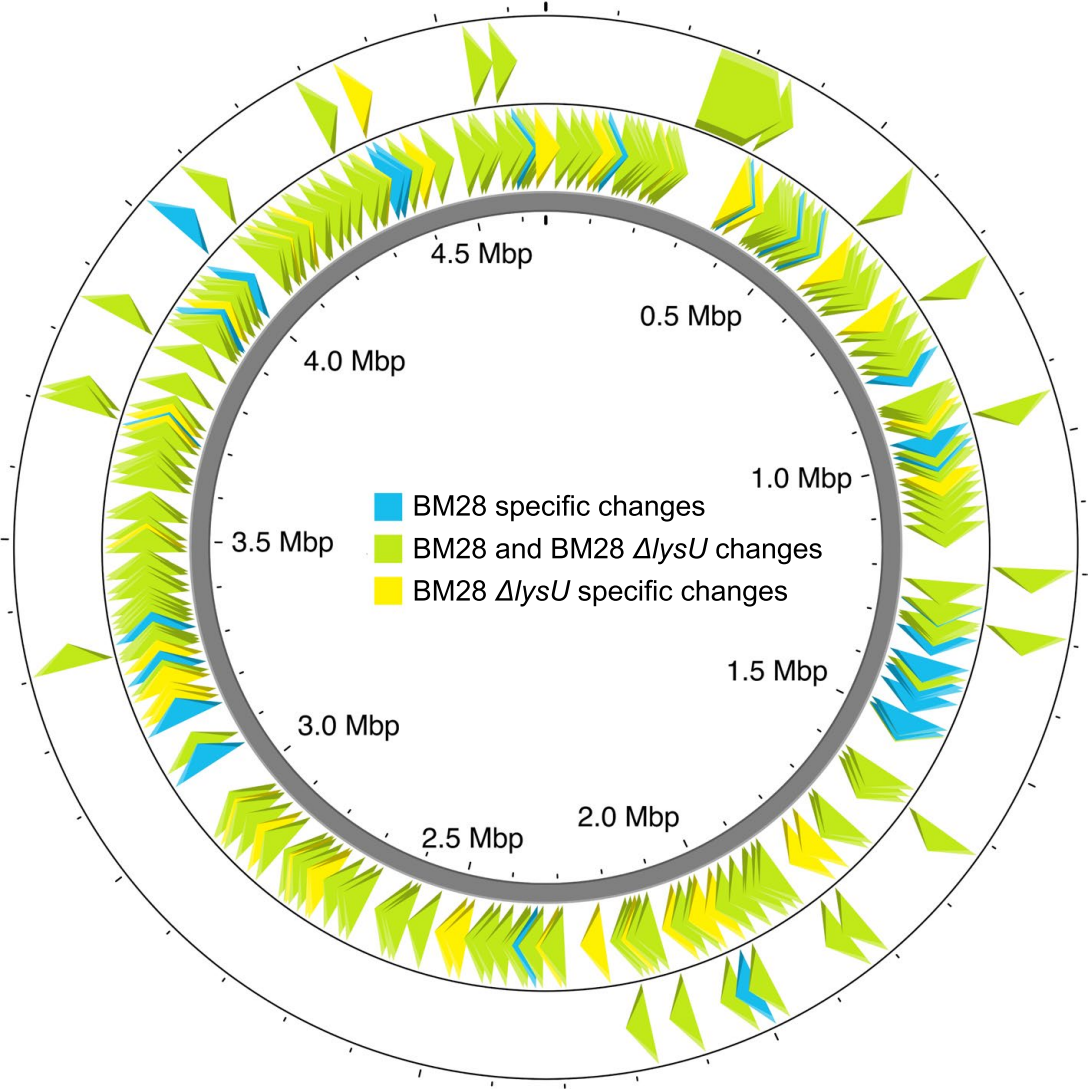
The changes in BM28 and BM28 *ΔlysU* compared to the reconstructed JB41 parent genome. The outer ring shows changes larger than 6 bp and the inner ring shows changes 6 bp or smaller. BM28 changes are shown as blue arrowheads, BM28 *ΔlysU* changes are shown as yellow arrowheads, and shared changes are shown as green arrowheads. Generated with Proksee [26]

Compared to MG1655 we found 233 changes in BM28 and 244 changes in BM28 *ΔlysU*, and compared to the inferred parent strain JB41 we found 231 and 242 changes in the strains, respectively (Fig. 2 and Supplementary Table S4). Thus, a majority of the remaining 231 or 242 changes in the strains likely arose throughout the heat evolution process. Dividing these numbers of changes by the length of the experiment (1,256 cultures subcultured every 48 h, thus, 2,512 days) results in 0.092 changes per day for BM28 and 0.096 changes per day for BM28 *ΔlysU* (Supplementary Table S4). Focussing in on the 151 BM28 SNPs and the 164 BM28 *ΔlysU* SNPs, mutational spectra were constructed (Supplementary Fig. S3). The most common base pair change, C-G —> T-A (a combination of C —> T and G —> A SNPs), accounted for 66% of all SNPs in BM28 and 62% of all SNPs in BM28 *ΔlysU*. Comparison of the BM28 and BM28 *ΔlysU* genomes revealed 75 differences, including the expected *lysU::cat* element for BM28 *ΔlysU* (Fig. 2 and Supplementary Fig. S4). Compared to MG1655 or JB41, BM28 has 32 of these differences and BM28 *ΔlysU* has 43 of them. Based on differences between the BM28 genome and the MC4100 *lysU::cat* genome used to delete *ΔlysU* from BM28 *ΔlysU*, it appears that about 55 kb (42–67 kb) of the MC4100 *lysU::cat* genome was swapped into the BM28 *ΔlysU* genome, including at least the region spanning the genes *yjdN* and *dcuA* (see Additional File S2 for more information). This P1 transduction event accounts for 6 of the 75 differences between BM28 and BM28 *ΔlysU*, adding 2 changes from the MC4100 *lysU::cat* genome and presumably removing 4 changes present in BM28 (Additional File S2). Excluding the variations coming from the MC4100 *lysU::cat* genome, there are 41 BM28 *ΔlysU* specific changes and 32 BM28 specific changes, for a total of 73 differences between BM28 and BM28 *ΔlysU* that arose over their evolution to high temperature. This leads us to believe that BM28 stocks were a population of genetically different cells, and thus the isolate chosen by Rudolph et al. for *lysU* deletion was genetically different than the BM28 isolate that we chose. Since the Winter group diluted their high temperature cultures 1:8 every 48 h, we think it is likely that the culture contained genetically distinct cells. With the average mutation rates (Supplementary Table S4; 0.092 changes per day for BM28 and 0.096 changes per day for BM28 *ΔlysU*) and numbers of strain specific mutations (32 BM28 specific changes and 41 BM28 *ΔlysU* specific changes) we estimate that the strains evolved separately for approximately the last year of the experiment (the calculation results in 348 days for BM28 and 426 days for BM28 *ΔlysU*). While these two strains were isolated from single colonies it is likely that a diversity of distinct strains co-evolved in the cultures.

### Mutation rates from stationary phase culturing of BM28 and BM28 *ΔlysU*

A variety of studies resulted in conflicting conclusions as to whether stressful growth conditions elevate the rate of spontaneous mutations. Rudolph et al. used stress-inducing stationary phase mutagenesis throughout the heat adaptation process, subculturing the cells every 48 h for 1,256 cultures (2,512 days). On average, excluding any changes that were introduced by the *lysU* deletion, this generated 0.092 and 0.096 changes in each 24-h period for BM28 and BM28 *ΔlysU*, respectively (Supplementary Table S4). *Escherichia coli* have spontaneous mutation rates of 10^–9^-10^–10^ changes per bp per generation [27], or 0.0046–0.00046 changes per genome per generation. Since the cultures were diluted 1:8, we can assume that the cells doubled approximately three times in the 48-h period, which is equal to 1.5 doublings per day. Assuming this and assuming a high spontaneous mutation rate of 10^–9^ changes per bp per generation, we would expect 0.0069 changes in every 24-h period. Based on this estimate, it appears that the BM28 strains were hyper-mutagenized over the course of their adaptation to heat, with estimated mutation rates ~ 13 X higher than would be expected based on our rough approximation.

### BM28 cells carry the *groESL* plasmid pOF39 but BM28 *ΔlysU* do not

**Fig. 3:**
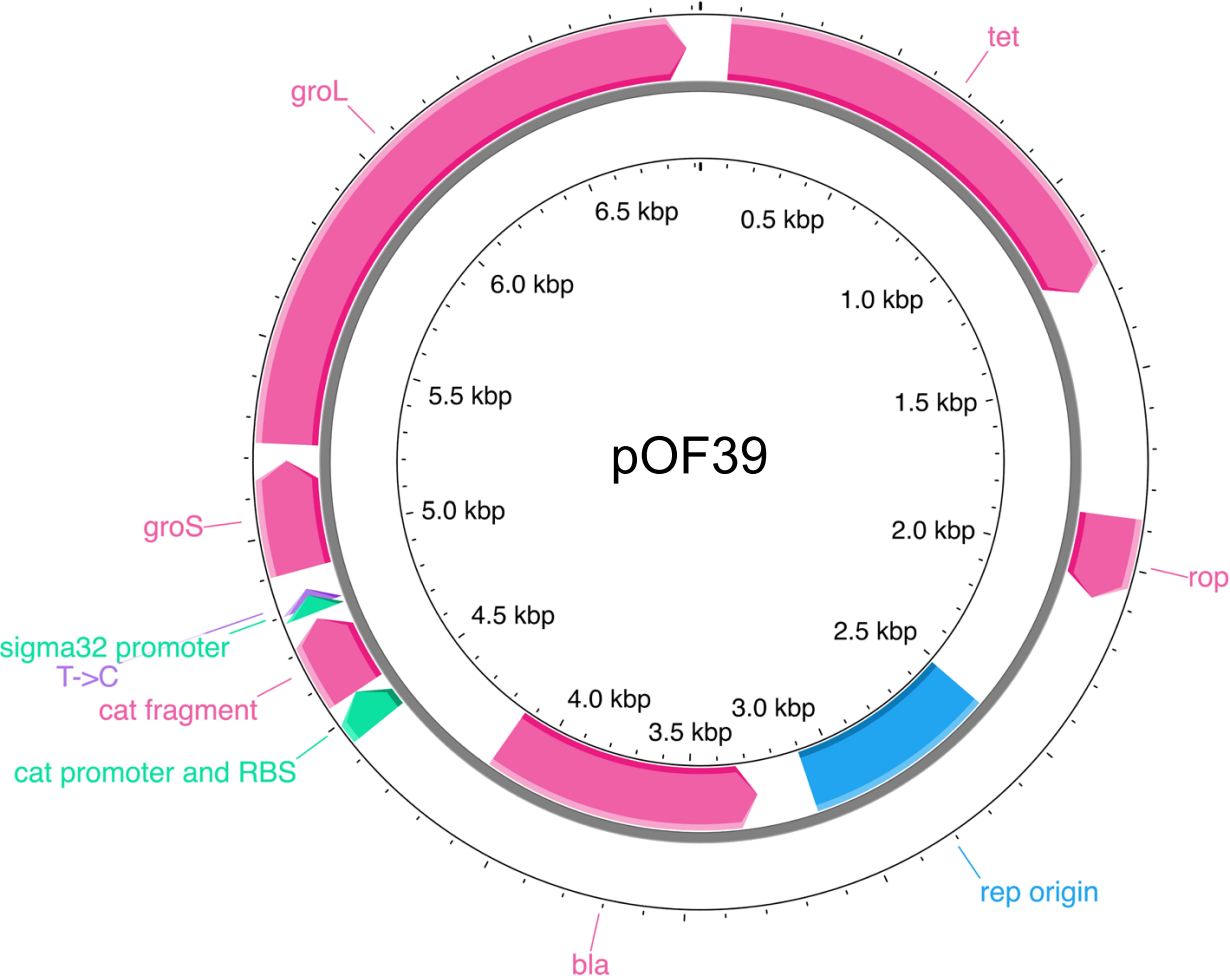
Map of the *groESL* plasmid pOF39, generated with Proksee [26]. Genes or gene fragments are shown in pink, regulatory elements in green, the *ori* in blue and the T- > C SNP in purple. This plasmid is found in BM28 and not in BM28 *ΔlysU*

In addition to the large BM28 SPAdes scaffold we found that corresponds to the chromosome, SPAdes built one more linear scaffold that was > 500 bp. This 4,875 bp scaffold aligned to various ColE1 family plasmids, containing the ColE1 *ori*, β-lactamase and a truncated chloramphenicol acetyltransferase gene. The scaffold had over 99% pairwise identity with pBR325 but did not appear to be a complete plasmid sequence. Oddities were also seen in the BM28 chromosome WGS data, such as a 2,065 bp region of high coverage spanning the *groESL* region which was surrounded by low frequency variations. Further exploration revealed that the missing part of the partial ColE1 plasmid sequence was the *E. coli groESL* region, and that the low frequency variations seen surrounding the chromosomal *groESL* region corresponded to sequences surrounding *groESL* in the plasmid (Fig. 3). We determined that this *groESL* plasmid was pOF39, generated by Fayet et al. [28], and we submitted the sequence to Genbank (Genbank OP156992.1).

In tracking down BM28, we were warned that the thermotolerant phenotype had been known to revert. Thus, we grew BM28 and BM28 *ΔlysU* at or above 42 °C to prepare glycerol stocks (46.8 °C) and for genomic DNA (gDNA) extractions (42–45 °C). From these glycerol stocks, we found that BM28 isolates carry pOF39 whereas BM28 *ΔlysU* isolates do not (Supplementary Fig. S5), and we also found no evidence of pOF39 in the BM28 *ΔlysU* Illumina WGS data. Chemical transformation of DH10B with BM28 gDNA preps yielded carbenicillin-resistant colonies (Supplementary Fig. S6) and a PCR of BM28 gDNA with pOF39 primers produced a product of the expected size (Supplementary Fig. S7).

We found a single T to C SNP in the pOF39 sequence, within the discriminator regions of the overlapping σ^32^ and σ^70^ *groESL* promoters (Additional File S2). Discriminators lie between the -10 motifs and the transcription start sites of promoters and some of the bases make contacts with the 1.2 regions of σ factors. This SNP slightly decreases the predicted transcription initiation rate of the σ^70^ promoter of *groESL* (from 1030 to 1003 au) by the De Novo DNA Promoter Calculator [29]. Consistent with the Rudolph et al. results, we found no mutation in the chromosomal *groESL* region of BM28. As well, the primers they used to look for mutations in the *groESL* region would not bind to the pOF39 *groESL* region, illustrating no discrepancies between ours and their results.

### pOF39 contributes to thermotolerance in BM28-related cells

**Fig. 4:**
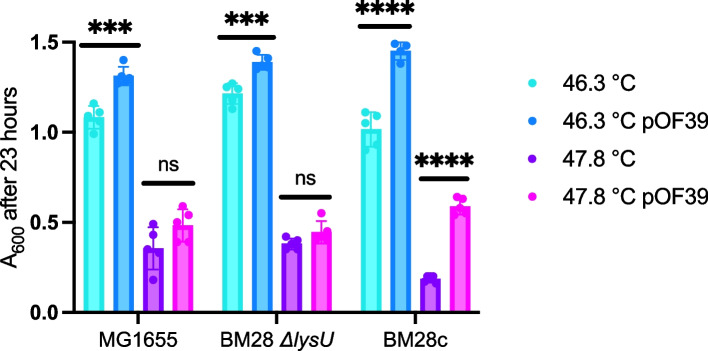
Final optical densities of MG1655, BM28 *ΔlysU* and BM28c (BM28 cured of pOF39) with and without pOF39 grown at high temperatures. Cultures were incubated at the indicated temperatures in a shaking water bath for 23 h, in quintuplicate, and their final optical densities were measured and recorded. Statistical comparisons between final optical densities of cells with and without pOF39 were determined using an unpaired t test with Welch correction, and *P* values > 0.05 are indicated with ns, ≤ 0.001 are indicated with ***, and ≤ 0.0001 are indicated with ****. Generated with GraphPad Prism 9.4.1

To explore the contribution of the *groESL* plasmid to the thermotolerance of BM28-related cells, we transformed pOF39 into BM28 *ΔlysU* and MG1655 and we cured some BM28 of pOF39 with sodium dodecyl sulphate (named BM28c for BM28 cured). We then carried out high temperature liquid growth experiments in the aforementioned strains with and without pOF39, at 46.3 °C and 47.8 °C (Fig. 4). At the lower temperature, all strains with pOF39 grow to significantly higher optical densities than the strains without the plasmid. At the higher temperature, only BM28 (with pOF39) grows to a significantly higher optical density compared to BM28 without pOF39 (BM28c). It should be noted that an increase in optical density does not necessarily indicate growth at that temperature. For example, the cells may grow at the very beginning of the experiment before the liquid media has reached the incubator temperature, and any inclusion body formation in the cells would also increase the optical density of the cultures [30]. Furthermore, the widely reported and accepted T_max_ of MG1655 in rich media grown aerobically is 45.5 °C, lower than both temperatures used in this experiment, and yet we still see an increase in the optical density over the course of the experiment. However, likely the same phenomenon has been described by the Van Impe group in a number of liquid culture studies [31–33], where they show a thermoresistant fraction of a population of MG1655 growing above their liquid T_max_ of 45.5 °C.

We also performed high temperature agar plate growth experiments in the strains with and without pOF39. On agar plates, there were larger differences between MG1655 and the BM28 strains in terms of growth at high temperature. On plates without antibiotics at 46.9–47.0 °C and 47.2–47.3 °C, pOF39 improved growth of all three strains (Supplementary Table S5). Importantly, pOF39 alone increased the T_max_ of MG1655 on an agar plate by at least 0.4 °C. In line with this, Rudolph et al. showed that a different *groESL* plasmid increased the T_max_ of their 37 °C-evolved strain in liquid media. On the other hand, BM28 and BM28 *ΔlysU* showed true growth at both high temperatures even without pOF39. This indicates to us that pOF39 is not the sole contributor to their increased thermotolerance, and that some of the chromosomal changes in the BM28 strains are critical to this phenotype.

### Expansion of IS10 elements, deletion of endogenous insertion sequences and small indels

As mentioned earlier, the BM28 strains carry the Tn10 transposon. Tn10 is a composite transposon flanked by two IS10s on either end; IS10L on the left side (further from *tetR*) and IS10R on the right side (closer to *tetR*) [34]. They differ by 19 bases, resulting in IS10L having very low activity due to changes in the promoter region. Analysis of the IS10s on the left and right side of the Tn10 in BM28 and BM28 *ΔlysU* revealed that the elements on the left were IS10R and the elements on the right were mutated IS10L/IS10R hybrids. Interestingly, the IS10L/R hybrids differ between BM28 and BM28 *ΔlysU* by four SNPs and small substitutions. In BM28, the IS10L/R element has essentially the IS10L promoter (Supplementary Fig. S8), whereas in BM28 *ΔlysU* the promoter is essentially IS10R for the first two thirds and IS10L for the last third (Supplementary Fig. S9). The last difference is in the IS10 transposase ORF: BM28 have a V301I mutation in their IS10L/R hybrid but BM28 *ΔlysU* lack this mutation. Aside from that difference, the IS10L/R hybrid ORFs are IS10L for the first third and IS10R for the last two thirds (Supplementary Figs. S8 and S9).

**Fig. 5:**
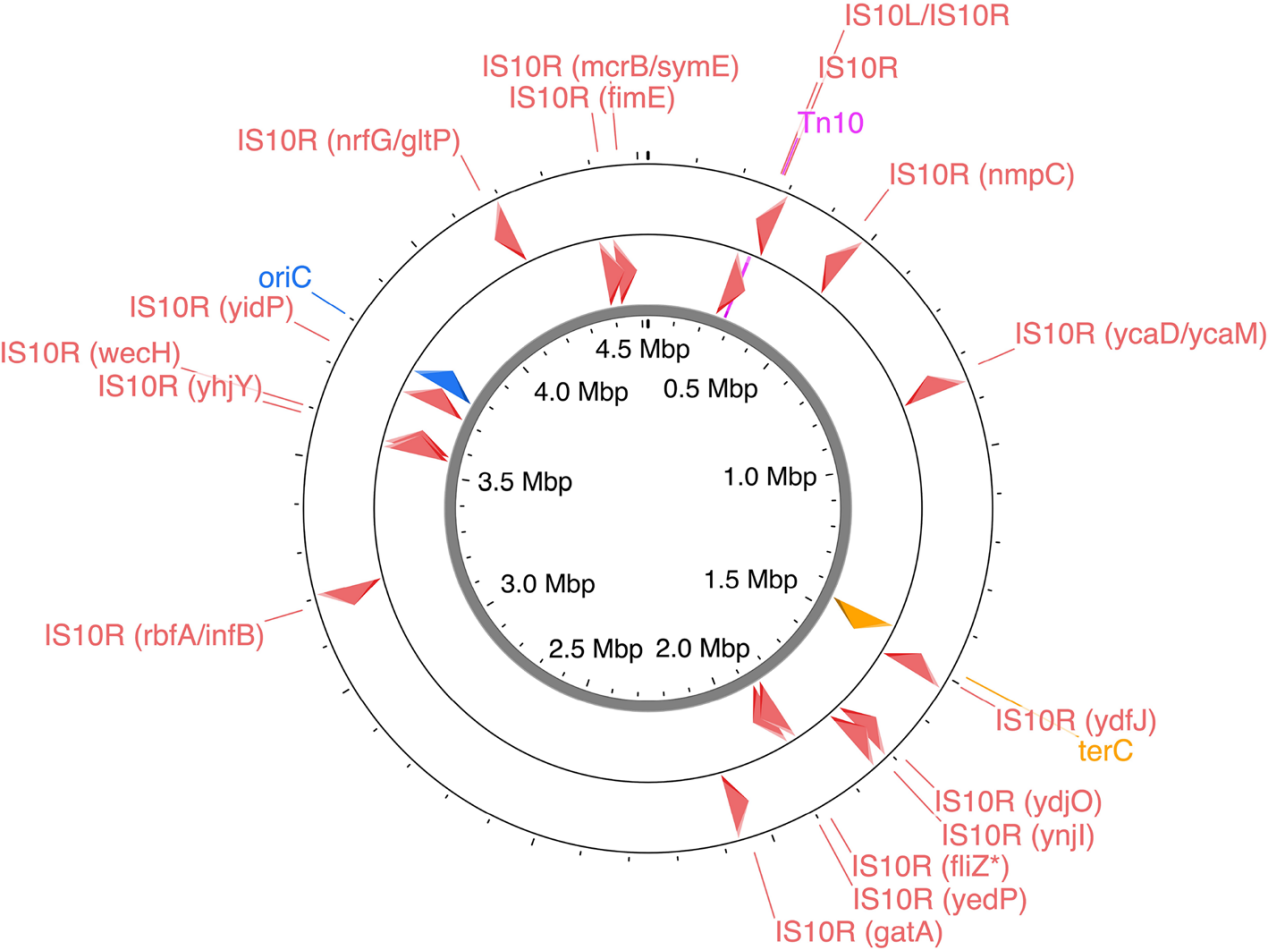
IS10R elements and the Tn10 transposon in the BM28 chromosome. A BM28 chromosome map with the positions of the IS10R elements and Tn10 transposon is shown, generated with Proksee [26]. The regions *oriC* and *terC* are also displayed. Compared to BM28, BM28 *ΔlysU* lacks the *fliZ* IS10R insertion

Remarkably, the BM28 genome contains a total of 17 IS10s, with 15 isolated IS10R copies (not part of the Tn10 transposon) inserted throughout the genome (Fig. 5). BM28 *ΔlysU* contains only 14 isolated IS10Rs, lacking the *fliZ*-interrupting insertion (Table 1 and Supplementary Fig. S4). IS10 elements operate via a cut-and-paste mechanism and generate a duplication of the 9 bp target sequence upon insertion, forming 9 bp direct repeats flanking themselves. For the 15 isolated BM28 IS10Rs, we analyzed the 9 bp target sequences they inserted into to ascertain the consensus sequence YRCTNNRNN, consistent with previous studies reporting the consensus sequence of the middle seven bases as GCTNAGC (Supplementary Fig. S10) [35]. Except for two elements in each strain, these IS10s are identical to the IS10R elements located in the left positions of the strains’ Tn10 transposons. The two nonidentical IS10s (interrupting *fimE* and inserting in between *mcrB* and *symE*) have a single A —> G silent mutation at nucleotide position 333 of the transposase gene, and they are relatively close to each other in the genome (38 kb apart). Based on their proximity and shared SNP, it is possible that one of these insertions gave rise to the other.

**Table 1:** IS10R insertions, frameshifts and truncations in BM28 and BM28 *ΔlysU*

Gene(s)^a^	B number(s)^b^	Gene product(s)	Change(s)^c^	Codon(s) affected / total codons^d^
*nmpC****#***	b0553	DLP12 prophage; putative outer membrane porin NmpC	I	**44/346**
*-ycaD-* > */ ****-ycaM-* > **	b0898/b0899	putative transporter YcaD / putative transporter YcaM	I	
*ydfJ*	b1543	putative transporter YdfJ	I	411/427
*ydjO*	b1730	protein YdjO	I	**175/267**
*ynjI*	b1762	DUF1266 domain-containing protein YnjI	I	**310/346**
fliZ*	b1921	DNA-binding transcriptional regulator FliZ	I	**12/183**
*yedP*	b1955	putative mannosyl-3-phosphoglycerate phosphatase	I	**105/271**
*gatA*	**b2094**	galactitol-specific PTS enzyme IIA component	I	**69/150**
** < *-rbfA-**** /* < *-infB-*	b3167/ **b3168**	30S ribosome binding factor / translation initiation factor IF-2	I	
*yhjY*	b3548	conserved protein YhjY	I	**158/232**
*wecH*	b3561	O-acetyltransferase WecH	I	**196/331**
*yidP*	b3684	putative DNA-binding transcriptional regulator YidP	I	**19/238**
*-nrfG-* > */ ****-gltP-* > **	b4076/ b4077	putative formate-dependent nitrite reductase complex subunit NrfG / glutamate/aspartate: H( +) symporter GltP	I	
*fimE*	b4313	regulator for fimA	I	**166/198**
** < *-mcrB-**** /* < *-symE-*	**b4346**/ b4347	5-methylcytosine-specific restriction enzyme subunit McrB / toxic protein SymE	I	
*caiT*	b0040	L-carnitine:gamma-butyrobetaine antiporter	T	**68/504**
*ampE*	b0111	protein AmpE	FS	**235/284**
*arfB*	b0191	peptidyl-tRNA hydrolase, ribosome rescue factor	FS	**103/140**
*mltD*	b0211	membrane-bound lytic murein transglycosylase D	FS	**110/452**
*fadE*	b0221	acyl-CoA dehydrogenase	FS	**611/814**
*mdlA*	b0448	ABC transporter family protein MdlA	FS	**234/590**
*ybbD****#***	b0501	putative uncharacterized protein YbbD	FS	139/86
*fepE*	b0587	polysaccharide co-polymerase family protein FepE	FS	**42/377**
*pagP*	b0622	Lipid IVA palmitoyltransferase	FS(79), T(112)	**79 and 112/186**
*galK*	b0757	galactokinase	FS	**271/382**
clsB*	b0789	cardiolipin synthase B	FS	**15/413**
*ybjL*	**b0847**	putative transport protein YbjL	FS	**81/561**
*potG*	b0855	putrescine ABC transporter ATP binding subunit	FS	**92/377**
*elfG*	b0941	putative fimbrial-like adhesin protein	FS	**140/356**
*narX*	b1222	sensory histidine kinase NarX	FS	586/598
*yciE*	b1257	DUF892 domain-containing protein YciE	T	**139/168**
recE*	b1350	Rac prophage; exonuclease VIII, ds DNA exonuclease, 5′ → 3’ specific	FS	**496/866**
ydaU*	b1359	Rac prophage; DUF1376 domain-containing protein YdaU	FS	**178/285**
*ydcO*	b1433	putative transport protein YdcO	FS	**256/391**
*lsrF*	b1517	3-hydroxy-2,4-pentadione 5-phosphate thiolase	FS	**21/291**
*marB*	b1532	multiple antibiotic resistance protein	T	**23/72**
*slyA*	**b1642**	DNA-binding transcriptional dual regulator SlyA	FS	**70/144**
*nimR*	b1790	DNA-binding transcriptional repressor NimR	FS	**174/273**
*cheR*	b1884	chemotaxis protein methyltransferase	FS	**221/286**
*fliR****‡***	b1950	flagellar biosynthesis protein FliR	FS (52.9%)	**67/261**
*gatC****#***	b2092	galactitol-specific PTS enzyme IIC component	FS	305/311
*menC*	b2261	o-succinylbenzoate synthase	FS	**15/320**
*evgS****‡***	b2370	sensory histidine kinase EvgS	FS	**315/1197**
*bcp*	**b2480**	thiol peroxidase	FS	**44/156**
*upp*	b2498	uracil phosphoribosyltransferase	FS	**147/208**
*ypjA*	b2647	adhesin-like autotransporter YpjA	FS	**112/1526**
*umpG*	b2744	broad specificity 5'(3')-nucleotidase and polyphosphatase	FS	**33/253**
*yggN*	**b2958**	putative EcfF	FS	**195/239**
*mtr*	b3161	tryptophan:H( +) symporter Mtr	FS	**335/414**
*glpR****#***	b3423	DNA-binding transcriptional repressor GlpR	FS	**51/105**
*pitA*	b3493	metal phosphate:H( +) symporter PitA	T	**385/499**
*gadW*	b3515	DNA-binding transcriptional dual regulator GadW	FS	**86/242**
*bcsB*	b3532	cellulose synthase periplasmic subunit	FS	**13/779**
*xylB*	b3564	xylulokinase	FS	**418/484**
*atpC*	b3731	ATP synthase F1 complex subunit epsilon	FS	136/139
*gpp*	b3779	guanosine-5'-triphosphate,3'-diphosphate phosphatase	FS	**387/494**
*cytR*	b3934	DNA-binding transcriptional repressor CytR	FS	**112/341**
yjdI*	b4126	PF06902 family protein YjdI	FS	**9/76**
cutA*	b4137	copper binding protein CutA	T	106/112
dcuA*	b4138	C4-dicarboxylate transporter DcuA	FS	**19/433**
*serB*	b4388	phosphoserine phosphatase	FS	311/322

^a^Arrows surrounding genes indicate their directions and genes in bold have the insertion upstream of their start codons, meaning the insertions may affect the promoters or ribosome-binding sites of the genes. Mutations only present in BM28 are indicated with an asterisk (*) and mutations only present in BM28 *ΔlysU* are indicated with a double dagger (‡). Genes that are already interrupted in the wildtype are marked with a number sign (#)

^b^The b number is shown in bold if the encoded protein was detected in an *E. coli* protein melting temperature study and determined to have a melting temperature ≤ 53.5 °C (≤ 5 °C above the T_max_ of BM28) [36]

^c^I represents an IS10R insertion, FS represents frame shift, T represents truncation, and if a change is at < 75% frequency the percent frequency is shown in brackets

^d^IS10R insertions, frame shifts and truncations which result in loss of > 10% of the amino acids are shown in bold

In addition to IS10 expansion, two endogenous insertion sequences were deleted in BM28 and BM28 *ΔlysU*: *insH21* between -*ychE-* > and -*oppA-* > and *insAB5* between < -*flhD-* and -*uspC-* > (Supplementary Table S6). Two small (~200 bp) repeat region deletions were found in BM28, likely arising due to homologous recombination between short repeats (Supplementary Table S6). Comparatively, BM28 *ΔlysU* lacks the repeat region deletion between *fre* and *fadA* (Supplementary Table S6 and Supplementary Fig. S4). Finally, a nearly 1300 bp deletion removed parts of the genes *ybfL* and *ybfD*, creating an in-frame YbfL-YbfD fusion protein-encoding gene (Supplementary Table S6). Since BM28 *ΔlysU* lacks the IS10 insertion in *fliZ* and the *fre/fadA* deletion, BM28 likely acquired these changes after the two strains diverged from a common ancestor (approximately 1 year prior to the end of the experiment). Building on this assumption, we can identify the other mutations specific to only one of the two strains as also occurring near the end of their evolutionary pathway (Additional File S2).

### BM28 and BM28 *ΔlysU* gene knockouts and mutations in essential and heat shock genes

To further explore BM28 and BM28 *ΔlysU* mutations, we analyzed the 43 BM28 and 41 BM28 *ΔlysU* genes frame shifted and/or truncated by SNPs and other small changes (Table 1). Amongst these knocked out genes, we found one putative (*yidP*) and four or five confirmed (*cytR, gadW, fliZ, nimR and slyA*, but *fliZ* is only interrupted in BM28) transcription factors in both (excluding the strains’ *glpR* interruptions because that gene is already interrupted in MG1655).

We next decided to look at classes of genes mutated in the BM28 strains more broadly. Using two *E. coli* essential gene studies, the Keio collection by the Wanner and Mori groups [37, 38] and the TraDIS study by Goodall et al. [39], we found that both strains have mutations in 15 essential genes, BM28 have intergenic mutations beside three essential genes and BM28 *ΔlysU* have intergenic mutations beside four essential genes (Table 2). In six of the ten essential genes with amino acid substitutions, these substitutions are predicted to increase the stability of the protein by DDGun [40] (Table 2). The remaining essential gene amino acid substitutions are either predicted to be neutral (two) or destabilizing (two). Though not technically/individually considered essential because there are seven copies of each gene, BM28 strains also have mutations in ribosomal RNA genes (Additional File S2). Using the list of heat shock genes determined by Nonaka et al. [41], we identified mutations in or around three heat shock genes for BM28 and two for BM28 *ΔlysU* (Table 3).

**Table 2:** BM28 and BM28 *ΔlysU* mutations in and around essential genes

Gene(s)^a^	B number(s)^b^	Gene product(s)	Mutation(s)^c^	ΔΔG predicted by DDGun [40] (kcal/mol)^d^
*tsf*	b0170	protein chain elongation factor EF-Ts	**Q114R**	0.1
** < *-proS‡-**** /* < *-trmO****‡****-*	b0194 / b0195	proline–tRNA ligase / tRNA m(6)t(6)A37 methyltransferase	A_8 _—> A_7_	n/a
*dxs*	b0420	1-deoxy-D-xylulose-5-phosphate synthase	**M159V**	-0.1
*hemH*	b0475	ferrochelatase	Silent codon 195	n/a
*sucA*	**b0726**	subunit of E1(0) component of 2-oxoglutarate dehydrogenase	**T434A**	0
*cydC*	b0886	glutathione/L-cysteine ABC exporter subunit CydC	**R183C**	0
*rpsA*	b0911	30S ribosomal subunit protein S1	Silent codon 73 (70.3%)	n/a
*mukB*	**b0924**	chromosome partitioning protein MukB	Silent codon 1374	n/a
** < *-asnS-**** /* < *-pncB-*	b0930 / **b0931**	asparagine–tRNA ligase / nicotinate phosphoribosyltransferase	A_7 _—> A_8_ (74.4%)	n/a
topA*	**b1274**	DNA topoisomerase 1	Silent codon 666	n/a
** < *-glyA-**** / ****-hmp-* > **	b2551 / b2552	serine hydroxymethyltransferase / nitric oxide dioxygenase	A_7 _—> A_6_	n/a
*alaS*	b2697	alanine-tRNA ligase/DNA-binding transcriptional repressor	Silent codon 196	n/a
*cca****‡***	b3056	fused tRNA nucleotidyltransferase/2',3'-cyclic phosphodiesterase/2' nucleotidase and phosphatase	Silent codon 345	n/a
** < *-rbfA-**** /* < *-infB-*	b3167 / **b3168**	30S ribosome binding factor / translation initiation factor IF-2	*tnpA* insertion	n/a
murA*	b3189	UDP-N-acetylglucosamine 1-carboxyvinyltransferase	**T10M**	0.4
*rpsH*	**b3306**	30S ribosomal subunit protein S8	**R13C**	-0.5
*rplC*	b3320	50S ribosomal subunit protein L3	**V189I**	0.1
*rho*	b3783	transcription termination factor Rho	**T96I**	0.6
*rpoC*	b3988	RNA polymerase subunit beta'	**A595V, T1135I**	0.6
*dnaB*	**b4052**	replicative DNA helicase	**P264S**	0.3

^a^For intergenic mutations, arrows surrounding genes indicate their directions, the essential gene in underlined and genes in bold have the insertion upstream of their start codons, meaning the insertions may affect the promoters or ribosome-binding sites of the genes. Mutations only present in BM28 are indicated with an asterisk (*) and mutations only present in BM28 *ΔlysU* are indicated with a double dagger (‡)

^b^The b number is shown in bold if the encoded protein was detected in an *E. coli* protein melting temperature study and determined to have a melting temperature ≤ 53.5 °C (≤ 5 °C above the T_max_ of BM28) [36]

^c^For coding mutations, nonsilent mutations are shown in bold and if a change is at < 75% frequency the percent frequency is shown in brackets

^d^The change in the Gibbs free energy of unfolding as predicted by DDGun [40] using the amino acid substitution(s) and a PDB structure or Alphafold predicted structure [42] of the protein. Positive values indicate an increase in protein stability and negative values indicate a decrease in protein stability

**Table 3:** BM28 and BM28 *ΔlysU* mutations in and around heat shock genes

Gene(s)^a^	B number(s)^b^	Gene product(s)	Mutation(s)^c^	ΔΔG predicted by DDGun [40] (kcal/mol)^d^
*ycjX*	b1321	DUF463 domain-containing protein YcjX	**R278C**	-0.8
topA*	**b1274**	DNA topoisomerase 1	Silent codon 666	n/a
** < *-hslV-**** /* < *-ftsN-*	b3932 / b3933	peptidase component of the HslVU protease / cell division protein FtsN	G_6 _—> G_4_	n/a

^a^For intergenic mutations, arrows surrounding genes indicate their directions, the heat shock gene in underlined and genes in bold have the insertion upstream of their start codons, meaning the insertions may affect the promoters or ribosome-binding sites of the genes. Mutations only present in BM28 are indicated with an asterisk (*) and there are no BM28 *ΔlysU* specific mutations

^b^The b number is shown in bold if the encoded protein was detected in an *E. coli* protein melting temperature study and determined to have a melting temperature ≤ 53.5 °C (≤ 5 °C above the T_max_ of BM28) [36]

^c^For coding mutations, nonsilent mutations are shown in bold and all changes are at 100% frequency

^d^The change in the Gibbs free energy of unfolding as predicted by DDGun [40] using the amino acid substitution(s) and a PDB structure or Alphafold predicted structure [42] of the protein. Positive values indicate an increase in protein stability and negative values indicate a decrease in protein stability

Lastly, we performed Gene Ontology and PANTHER analyses [43–45] to determine whether specific genes or intergenic regions near specific genes were more or less often mutated than expected (Supplementary Tables S7 and S8). When we included all genes with changes and genes surrounding intergenic regions with changes, including those that were part of deletions, there were no statistically significant results, indicating that gene regions associated with particular biological processes, molecular functions, cellular components, pathways, etc. were not enriched or de-enriched in mutations in BM28 nor BM28 *ΔlysU*. However, if we excluded the genes that were completely deleted, we saw an overrepresentation of changes in or surrounding genes with the cellular components membrane (GO:0,016,020) and cellular anatomical entity (GO:0,110,165), and for BM28 *ΔlysU* only, integral component of membrane (GO:0,016,021) (Supplementary Tables S7 and S8). For both strains we also found an underrepresentation of genes with the cellular component unclassified (UNCLASSIFIED) with intragenic or nearby intergenic changes (Supplementary Tables S7 and S8). Thus, excluding completely deleted genes, genes encoding membrane-associated proteins and proteins that are part of something larger than a protein complex are enriched in intragenic and intergenic changes, whereas genes encoding proteins with unclassified cellular components are de-enriched in intragenic and intergenic changes.

SNPs and small indels in and around genes which were neither essential nor heat-induced are listed in Additional File S2 alongside all of the genetic changes in the strains. One such mutation to note present in both strains changed the start codon of *flgA* from AUG to AUA, which when used as a start codon for GFP decreases the predicted translation rate by over 100 X [46]. The codon 3’ to the wildtype AUG is a CUG, which could also serve as a start codon, but CUG is comparable to AUA in terms of its translation initiation rate in the same GFP study. FlgA helps assemble the P-ring of flagella and its deletion renders cells nonmotile. Indeed, a soft agar motility assay showed that unlike DH10B and MG1655, BM28 and BM28 *ΔlysU* are nonmotile (Supplementary Table S9).

## Discussion

### Potential mechanism for the large chromosomal deletion

One surprising finding was the 123 kb deletion that fused *dinB* and *mhpE* (Fig. 1 and Supplementary Table S3). Several insertion sequences reside in the deleted region, and there were clearly insertion sequence transposition events happening throughout the strains’ evolution to heat. In particular, IS10 transposition events cause double-stranded breaks through their cut-and-paste or nonreplicative transposition mechanism. In response to a double-stranded break in the chromosome, *E. coli* have an interesting alternate end joining DNA repair mechanism that relies on (sometimes excessive) DNA resection and ligation to close the chromosome at microhomologous sites [47]. *dinB* and *mhpE* share 12 bp of identical sequence (Supplementary Fig. S1), which appear to have served as microhomologous sites to close the chromosome. Whether through insertion sequence transposition(s) or other means, it is likely that a double-stranded break occurred in the 123 kb deleted region and in response, the chromosome was repaired via the alternate end joining mechanism.

### Possible sources of plasmid pOF39

The discovery of the presence of plasmid pOF39 begs the question: when and how was it introduced? One possibility is that the plasmid was introduced into the lineage between the BM16 and BM25 isolates, that were evolved at 45 °C and 48 °C, respectively. This ideas is consistent with the 2D gel data, the comment by the authors that BM16 and later isolates fail to grow above 45 °C after high dilutions and the difficulty the BM16 lineage had in making the transition from growth at 45 °C to growth at 47 °C. Plasmid pOF39 was not selected for in the typical and stringent manner of antibiotic selection, but was instead likely maintained because it was beneficial for growth at high temperature. Therefore, it may be that high dilutions lead to complete loss of the plasmid, preventing cell growth at higher temperatures in some lineages. Importantly, Rudolph et al. showed that after a heat shock at 49 °C for 3 h, BM25 shows ~ 5000 X higher viability than BM16. This could be in large part due to the acquisition of pOF39. Alternatively, it is possible that the starting strain contained the pOF39 plasmid, and that a combination of genetic changes and selective pressure at higher incubation temperatures lead to a large increase in GroESL expression levels between isolates BM16 and BM25. The attractiveness of this idea is that it does not require an explanation of how plasmid pOF39 was taken up by *E. coli*, which is not naturally transformable.

### The differences between BM28 and BM28 *ΔlysU* can be explained by the loss of pOF39

Rudolph et al. observed differences in GroESL levels and viability to heat shock after deletion of *lysU*, which they attributed to loss of *lysU*. However, we postulate that these differences are caused by the presence of pOF39 in BM28 and the absence of pOF39 in BM28 *ΔlysU*. We suspect that the strain that went on to become BM28 *ΔlysU* was cured of its pOF39 during the P1 transduction process to delete *lysU*. Taking into consideration the BM28 thermotolerance reversion warning and the Winter group’s data showing that BM28 is much less fit at 37 °C compared to the 37 °C-evolved strain and earlier heat evolved isolates, we believe that it is beneficial for cells to lose pOF39 when incubated at moderate temperatures, such as those used in P1 transduction experiments. Thus, we believe that like BM28, the strain that went on to become BM28 *ΔlysU* likely carried pOF39 at the end of its evolution but lost it in the P1 transduction process.

### IS10R expansion theories and possible effects of IS10R insertion

Other unexpected findings were the extra 14–15 copies of IS10 scattered throughout the genomes. IS10s are non-replicative transposons, operating via a cut-and-paste mechanism. Thus, it is curious that a IS10 expansion event seems to have occurred in the BM28 strains, interrupting 10–11 genes and inserting into four intergenic regions (Fig. 5 and Table 1). However, non-replicative insertion sequences do increase in copy number in some descendants due to specific cut-and-paste events occurring during genome replication, and homologous recombination may play an additional role in some cases [48]. A study of the insertion sequence family IS4, which IS10s belong to, showed that substantial IS4 expansions events have occurred in some pathogens and extremophiles [49]. IS10 transposition is repressed at multiple levels and IS10 elements display multicopy suppression where the transposition activity decreases with increasing copies of Tn10/IS10 [50]. However, elevated temperatures [51], stationary phase culturing [52] and specific hemimethylation states of the DNA [53] can increase their transposition activity.

We believe that the insertion into the highly expressed *metY-pnp* operon (between *infB* and *rbfA*) is likely a contributor to the IS10R expansion (Fig. 5 and Table 1). *metY-pnp* is an eight gene operon which is transcribed into a number of polycistronic mRNAs. Three of the eight genes are clearly essential (*nusA, infB, rpsO*), and most of the products of these genes are present in the thousands per cell in MG1655 grown in complete media [54]. IS10R is inserted in the same orientation as the operon and would likely be present on a number of the operon’s polycistronic mRNAs. All together we think this IS10R insertion has the most potential for high transposase expression, and because of this we speculate that this IS10R may have been one of the first IS10R insertions in the strain, driving further insertions.

### Noteworthy mutations in BM28 and BM28 *ΔlysU*

Through a combination of frame shifts, truncations, insertion sequence transpositions and large deletions, 223 genes were knocked out in BM28 and 220 genes were knocked out in BM28 *ΔlysU* (Supplementary Table S3 and Table 1). For 56 of these 223 BM28 genes, the melting temperatures of the proteins are known [36]. Fourteen of these 56 genes (25%) encode proteins with melting temperatures ≤ 5 °C above the T_max_ of BM28 (≤ 53.5 °C) [36]. This proportion of the encoded proteins with melting temperatures ≤ 53.5 °C is comparable to the proportion of *E. coli* protein melting temperatures ≤ 53.5 °C determined by Mateus et al. (21.9%), thus, genes encoding low melting temperature proteins were not preferentially deleted in the BM28 strains [36]. Even so, it could be adaptive to delete nonessential genes encoding low melting temperature proteins if they are being expressed, to reduce protein unfolding or misfolding and subsequent protein aggregation in the cell at high incubation temperatures. An additional 14 BM28 and 15 BM28 *ΔlysU* genes encoding proteins with low melting temperatures had missense mutations (Additional File S2). When we ran these missense mutations together with published structures or Alphafold predicted structures [42] of the proteins through DDGun [40], the web-server predicted that six of the mutations increased protein stability, six of the mutations decreased stability and the remaining two BM28 and three BM28 *ΔlysU* mutations had no effect on stability (Additional File S2). When we performed the same analysis on missense mutations where the encoded protein melting temperatures were > 53.5 °C, we saw a similar distribution of predictions (18 stabilizing, 16 destabilizing and 9 neutral for BM28 and 17 stabilizing, 16 destabilizing and 8 neutral for BM28 *ΔlysU*). The strains also had changes in and around essential genes and heat shock genes (Tables 2 and 3), some of which (6 of 11 for BM28 and 5 of 10 for BM28 *ΔlysU*) were predicted to increase the stability of the proteins, which could clearly be helpful for growth at high temperature. Finally, a number of missense and silent mutations occurred in nonessential genes that are not heat induced (Additional File S2). It is important to note that even synonymous mutations can have substantial effects on proteins and organisms, including changes in substrate specificity [55], improved replication of a virus at high temperatures [56] and changes in organism fitness [57–61]. We will discuss select mutations that seem potentially important to growth at high temperature below.

#### Master regulators and transcription factors

Amongst the 223 or 220 inactivated genes are five confirmed transcription factors: *cytR, gadW, fliZ* (in BM28 only), *nimR* and *slyA*, which regulate 13, 15, 21, 2 and 37 genes, respectively [62]. These knockouts likely rewire transcription in BM28 and BM28 *ΔlysU* to some extent. Other transcription related proteins mutated in the strains include master regulators. Previous ALE studies, whether to heat or other stressors, identified changes in master regulators of transcription such as Rho and RpoB [14–17]. Changes in these master regulators have been shown to change the expression of thousands of genes, and thus changes in these regulators can lead to adaptive advantages in the presence of various stressors [16, 17]. In many of these studies, changes in *rpoB, rho* or other genes shift gene expression patterns back towards an unstressed state despite the cells being in the stress condition [16–18]. BM28 and BM28 *ΔlysU* have changes in Rho (T96I) and in the RNA polymerase beta’ subunit RpoC (A595V and T1135I), which very likely cause changes in gene expression. These strains also have a substitution (D90N) in the RNAP-binding protein DksA which is a major player in the stringent response. All of these master regulator amino acid substitutions are predicted to be stabilizing by DDGun [40], except for RpoC T1135I which is predicted to be neutral (Table 2 and Additional File S2).

T96 in Rho resides on the surface and does not appear to contact the RNA polymerase (RNAP) nor fellow Rho monomers in the Rho hexamer in the RNAP-Rho pretermination complex (PDB 6XAS, see Supplementary Fig. S11) [63]. A595 in RpoC is also on the surface of the protein and does not seem to contact other RNA polymerase components nor Rho nor DksA in the RNAP-Rho (PDB 6XAS, see Supplementary Fig. S12) and RNAP-DksA-ppGpp (PDB 5VSW, see Supplementary Fig. S12) [64] complexes, though it is fairly near to where DksA binds RpoC and RpoB (the closest distance between RpoC A595 and a DksA residue, D64, is 16.6 Å, see Supplementary Fig. S12). RpoC T1135 is in close proximity to DksA (closest distance 7.1 Å to DksA D90, see Supplementary Fig. S13) and one of the ppGpps which bind DksA and RpoC (closest distance 13.4 Å, see Supplementary Fig. S13). RpoC T1135, DksA and that ppGpp molecule are also in close proximity to the active site magnesium ion in the RNAP-DksA-ppGpp structure, which is near to the DNA in promoter open complex RNAP structures (PDB 6OUL, see Supplementary Fig. S14) [65]. It is interesting that both RpoC T1135I and DksA D90N substitutions occurred, because these residues are quite close to each other in RNAP-DksA complexes. It is tempting to speculate that these two mutations are related in some way, perhaps through one arising first and the other arising in response. DksA binds the RNA polymerase, and that binding alone or that binding plus binding of the stress alarmones pppGpp or ppGpp (abbreviated together as (p)ppGpp) especially, leads to inhibition of transcription from promoters which form intrinsically unstable open complexes, such as those controlling ribosomal genes. In a study that searched for changes in DksA which enable it to function well without (p)ppGpp [66], the authors found a significant mutation, N88I, that is just two amino acids N-terminal to D90. It is possible that the D90N mutation in BM28 strains modulates the function of DksA or binding affinity of DksA for RNAP.

#### (p)ppGpp and polyphosphate

A few other mutated genes in BM28 and BM28 Δ*lysU* have connections to (p)ppGpp and to polyphosphate. (p)ppGpp and polyphosphate levels increase in response to stress, and polyphosphate has ATP-independent molecular chaperone activity [67]. The *gpp* and *umpG* (also known as *surE*) genes were inactivated by frame shift mutations. The proteins encoded by these genes have polyphosphatase activity, suggesting that their knockouts may reduce the degradation of polyphosphate in the cells. ppGpp and pppGpp especially inhibit polyphosphate degradation by the exopolyphosphatase Ppx, and Gpp also converts pppGpp to ppGpp. The *gpp* frame shift is far enough into the gene that the encoded protein retains the domains required for conversion of pppGpp to ppGpp and for polyphosphatase activity (frame shifted after amino acid 387 of 494) [68, 69]. However, the frameshift occurs partway through one of the C-terminal domains, indicating that this frameshift could cause abnormal folding and therefore perhaps degradation of Gpp. Whether or not functional Gpp is made in BM28 strains, the *umpG* frame shift clearly deletes the protein. Thus, there may be a higher level of polyphosphate in the cells, which would likely be beneficial for high temperature growth.

#### Heat shock related

The BM28 and BM28 *ΔlysU* strains have a few heat shock related mutations which may be important to their thermotolerant phenotypes. In both strains, *cytR* has a frame shift mutation starting at codon 112 of 341. Though *cytR* is not a heat shock gene, it is a transcriptional repressor of *rpoH*: the heat shock σ factor σ^32^. While this frame shift truncates the protein after its DNA-binding domain, CytR relies on binding another transcriptional regulator, CRP, in order to bind DNA well [70]. The frameshift in these strains removes CytR’s CRP-binding region, leaving CytR impaired in its DNA binding. Thus, this frame shift mutation likely has a similar effect to total deletion of *cytR*, which has been shown to lead to an increase in *rpoH* transcription using *rpoH-lacZ* transcriptional fusions [71, 72]. Since *rpoH* is under many levels of negative regulation, the effect of an increase in its transcription is unclear. At low to moderate temperatures, the ribosome-binding site of *rpoH*’s mRNA is occluded, leading to low levels of translation. When translation does occur, many heat shock proteins directly or indirectly contribute to σ^32^’s degradation or inactivation if they are not otherwise occupied with unfolded or misfolded proteins, effectively turning down the heat shock response when it is at a higher level than required.

Another heat shock related mutation that BM28 and BM28 *ΔlysU* share is a two bp deletion within the σ^32^ promoter of *hslVU*. Two C’s are deleted from a run of seven C’s present upstream and within the -10 region of the σ^32^ promoter. From the perspective of maintaining the same -10 region which matches well with the σ^32^ consensus sequence [41], this change effectively decreases the number of bases between the -35 and -10 regions from 14 to 12, which changes this distance from the most common to one not seen among the 50 σ^32^ promoters studied by Nonaka et al. [41]. However, the De Novo DNA Promoter Calculator predicts that this two bp deletion increases the predicted strengths of potential σ^70^ promoters around this region (~55 bp upstream of the *hslV* start codon, from strengths of ~2000 to ~6700 au) [29]. If we compare the wildtype σ^32^-dependent transcript to the potential σ^70^-dependent transcript, this two bp deletion is also predicted to increase the translation initiation rate of *hslV* from 1883 to 2306 au by the ribosome-binding site Calculator [73]. Thus, it may be that *hslVU* shifts away from σ^32^ regulation and towards σ^70^ regulation in these strains, and the new transcriptional start site may boost the translation initiation rate of *hslV* and possibly *hslU* through translational coupling [74]. It is possible that *hslVU* would be expressed at higher levels than usual at moderate temperatures and at lower levels than usual at higher temperatures, for a more consistent expression level over a variety of temperatures. As well, perhaps this shift is also important in terms of avoiding the negative feedback regulation of the heat shock response. For example, when *E. coli* are shifted from 30 °C to 42 °C, σ^32^ levels increase rapidly and then decrease to a new steady state level higher than the steady state level at 30 °C [75]. The levels and activities of the heat shock proteins affect the levels and activities of the other heat shock proteins, and thus a change to σ^70^-dependent expression should bypass this negative feedback.

There is considerable evidence that the HslVU protein complex plays an important role in adaptation to high temperature growth. Mutations in *hslVU* have been shown to increase the T_max_ in minimal media [76], impair growth on rich media plates at high temperatures [77], not impair high temperature rich media plate growth [78, 79] and arise mostly when adapting to 37 °C and not higher temperatures [80]. Mutations in this operon have also been shown to change the expression of hundreds of genes [76], lead to small cells in minimal media but not rich media [77], not significantly change cell size in minimal media [81], and lead to changes in the timing of chromosome separation and/or cell constriction [81]. One unresolved issue surrounding HslVU is whether or not it degrades σ^32^ in vivo. Though HslVU has been shown to degrade σ^32^ in vitro [82], we could not find evidence supporting HslVU as a major degrader of σ^32^ in vivo. Additionally, in vitro experiments and yeast two-hybrid studies do not detect interactions between HslU and σ^32^ [83, 84] nor between HslV and σ^32^ [83]. Although many authors contend that HslVU degrades σ^32^ in vivo, citing studies from the Yura group, these researchers had merely posited that while that HslVU can degrade σ^32^ in vitro [82], in vivo HslVU may degrade σ^32^ but that a HslVU knockout or overexpression could also affect σ^32^ indirectly [85]. Importantly, Dr. Yura states in a 2019 review article [86] that HslVU *may* degrade σ^32^ in vivo. Complicating any interpretation is the possibility that HslVU specifically degrades σ^32^ in vivo at elevated temperatures, and many of these studies were performed at lower temperatures.

The HslVU complex is interesting because though the complex contains 12 of each protein, the number of HslU proteins in the cell is about 1.5 times that of the number of HslV proteins in the cell when grown in multiple different media [54]. In HslVU the HslV hexamer rings are in the middle and the HslU hexamer rings are on the ends, sandwiching the HslV hexamers [87]. Since it is HslU which first contacts client proteins, and therefore determines the substrates of HslVU, the extra HslU may act as a chaperone for the same substrates that HslVU degrades. This seems to be the case for the DNA damage induced cell division inhibitor protein SulA, where HslU helps SulA to properly fold while HslVU degrades SulA [88, 89]. However, the Lon protease seems to be the primary protease for SulA. Like SulA, many proteins can be degraded by multiple proteases in the cell, and this could be related to the different melting temperatures and optimal temperatures of these proteases. For instance, both Lon and ClpX have melting temperatures of 51.1 °C [36] whereas HslVU is most active around 55 °C [90], suggesting that for some substrates HslVU may take over for Lon and ClpPX at high temperatures.

Altogether, these studies highlight the importance of the experimental conditions and genetic backgrounds on whether or not mutations are adaptive. On the one hand, *hslVU* could be dispensable for high temperature growth in minimal media because the T_max_ in minimal media is much lower than in media with methionine, and thus other more thermosensitive proteases maintain their activity. In those cases, it is possible that nonsense and missense mutations in *hslV* or *hslU* increase the level of misfolded and unfolded proteins in the cell, titrating heat shock proteins away from inactivating and/or degrading σ^32^ and thus ramping up the heat shock response. There has also been work showing that cells which inherit protein aggregates have higher viability to heat shock than cells that do not, likely due to inheriting more chaperones and proteases associated with the aggregates [91]. However, we must consider the tremendous distinction between survival to heat shock above the T_max_ and what we are more interested in in heat ALE studies: growth at temperatures below or at the T_max_ [91]. In BM28 and BM28 *ΔlysU*, especially near the rich liquid media T_max_ of 48.5 °C, it could be that HslVU activity is essential for growth because the cells require an active protease for survival. HslVU’s presence could also be important due to its relationship with SulA, as we suspect that SulA and other SOS response proteins were expressed frequently in BM28 strains due to the IS10 insertions and large chromosomal deletions. If a shift away from σ^32^ and towards σ^70^ regulation leads to a more consistent expression of HslVU across many temperatures, perhaps this is adaptive for cells experiencing periodic DNA damage and higher incubation temperatures.

## Conclusions

It is clear that BM28 strains followed interesting and unique evolutionary routes to their impressive T_max_’s. Chromosomal changes and the *groESL* plasmid pOF39 both seem to have contributed to their thermotolerance. Without pOF39, both BM28 and BM28 *ΔlysU* are still thermotolerant, but pOF39 was probably important for the strains during their evolution and it certainly contributes to their heat tolerance [9]. On the chromosomal side, we suspect that the master regulator and *hslVU* mutations are important to the thermotolerant phenotype. As well, we were able to clarify two points the Rudolph et al. paper is commonly cited for through WGS of the strains, without which we would also come to the original authors’ conclusions. Both centre around the *groESL* plasmid pOF39; BM28 overexpress GroESL due to the plasmid and not due to ALE to heat, and LysU does not seem to be important to thermotolerance in BM28 strains, rather, the pOF39 plasmid is. It will be interesting to examine other evolutionary routes towards increased thermotolerance in *E. coli* in future studies and to compare them with the few studies that have been published to date. In the future it is likely that researchers in this field will uncover more distinct routes to thermotolerance in *E. coli* and be able to classify the routes into general categories, to ultimately define how a bacterium can evolve to grow at higher temperatures. Bettering our understanding of enhancing thermotolerance in bacteria may have applications in enhancing thermotolerance in more complex organisms, especially those most affected by climate change and those important to humans or other organisms.

## Experimental procedures

### gDNA extraction and whole-genome sequencing

20–25 mL LB (1% w/v tryptone, 0.5% w/v yeast extract, 0.5% sodium chloride) cultures of BM28 and BM28 *ΔlysU* were grown up at 200–250 rpm overnight at 42–45 °C. Cells were harvested from the cultures, suspended in TE buffer (10 mM Tris, 1 mM EDTA), and lysed with proteases (Proteinase K or subtilisin) and 0.2–0.5% w/v sodium dodecyl sulphate at 37–50 °C. gDNA was extracted with 25:24:1 phenol:chloroform:isoamyl alcohol or guanidinium hydrochloride and ethanol, followed by cleanup on a silica column and elution in 10% v/v TE buffer (1 mM Tris, 0.1 mM EDTA). gDNA samples were sent to the Microbial Genome Sequencing Center in Pittsburgh for Illumina and Oxford Nanopore sequencing. For Illumina sequencing, 150 Mb sequencing packages were purchased for each sample (guaranteeing 32 X coverage for MG1655-derived BM28 and BM28 *ΔlysU*) and for Nanopore sequencing, Nanopore Only sequencing packages were purchased for each sample, which guaranteed a minimum of 300 Mb of sequencing data per sample (64 X coverage for MG1655-derived BM28).

### Whole-genome sequencing data processing and analysis

For Illumina data, FastQ paired end read files were imported into Geneious using the default settings and Geneious automatically determined the read technology, so the only setting changed or inputted was that the insert size was set to 500 bp. Reads were trimmed using BBDuk with “trim adapters” selected with the default settings, “trim low quality” set to “both ends” with a minimum quality of 30, “trim adapters based on paired read overhangs” set to a minimum overlap of 24 and “discard short reads” set to a minimum length of 30. The reads were not normalized. The trimmed reads were mapped to the MG1655 reference genome or the JB41 draft genome depending on the purpose, using the default settings of map to reference with “do not trim” selected. Using the contig generated by mapping the trimmed paired end reads to the JB41 draft genome reference, we found variations/SNPs using the default settings of “find variations/SNPs”. The variations/SNPs details were exported from Geneious Prime and analyzed in Microsoft Excel. For Nanopore data, FastQ files were imported into Geneious Prime and “Nanopore” was selected as the data type. Q30 trimmed Illumina paired end reads and raw nanopore reads were used for a SPAdes de novo assembly. The data source was set to “Multi Cell”, the method to “Assemble”, and the “Careful Mode” and “Do Not Trim” options were used. We compared the BM28 and BM28 *ΔlysU* genomes to the MG1655 reference genome with the progressive Mauve algorithm [23] set to default settings.

### Identifying pOF39 in BM28, curing BM28 of pOF39 and transforming cells with pOF39

To initially detect pOF39 in BM28, extracted BM28 gDNA was transformed into chemically competent DH10B and the transformation was plated onto 1.5% w/v agar LB + Cb (100 µg/mL carbenicillin) plates. As well, primers specific to pOF39 sequences surrounding the *groESL* insert (forward primer: TTCAGCTGGATATTACGGCC, reverse primer: TGAGCGCATTGTTAGATTTCATAC) were used in a PCR with extracted BM28 gDNA as the template. BM28 and BM28 *ΔlysU* were plated from glycerol stocks onto LB and LB + Cb agar plates, incubated overnight at 37 °C, and growth on both plates were compared. A colony of BM28 from LB agar was subcultured in LB broth with 1% w/v sodium dodecyl sulphate every 24 h for three days, using a 100 X dilution. After the three days, cells were plated from the culture on LB agar and the plate was incubated at 37 °C overnight. The next day, single colonies were plated onto LB and LB + Cb agar and incubated at 37 °C overnight. Isolates which grew on LB but did not grow on LB + Cb were saved as presumptive pOF39-cured BM28. Plasmid DNA was prepared from BM28 that had been confirmed to carry pOF39, and the isolated plasmid DNA was used to transform MG1655 and BM28 *ΔlysU*.

### High temperature growth experiments

MG1655, BM28 *ΔlysU* and BM28c (BM28 cured of pOF39) with and without pOF39, were plated at 37 °C on LB + Cb and LB plates, respectively. The next day, 2 mL LB aliquots in 16 mm glass test tubes were inoculated with several colonies of each sample, in quintuplicate, and incubated with shaking at 250 rpm in a water bath. Twenty-three hours later the final optical densities were measured and recorded. The experiment was performed at 46.3 °C and 47.8 °C. For high temperature plate growth experiments, the six isolates were plated for single colony isolation onto LB plates at 46.9–47.0 and 47.2–47.3 °C. Forty-eight hours later, plates were removed from the incubators and the isolates were scored for growth.

### Motility assays

DH10B, MG1655, BM28 and BM28 *ΔlysU* (all lacking pOF39 except for BM28) were plated onto LB agar and incubated at 37 °C overnight. A single colony for each strain was picked with a sterile wooden stick and stabbed about two thirds of the way into the centre of 5 mL of soft LB agar (0.35% w/v agar) in a glass test tube and incubated overnight at 37 °C. The next day, the tubes were inspected for growth and scored as nonmotile if they grew at the edges of the stab and scored as motile if they grew throughout the soft agar. The experiment was performed in duplicate.

## Supplementary Information

**Additional file 1: Supplementary Table S1.** BM28 and BM28 *ΔlysU* NextSeq 2000 Illumina WGS data statistics. **Supplementary Table S2.** BM28 Oxford Nanopore WGS data statistics. **Supplementary Table S3.** Large deletions in BM28 and BM28 *ΔlysU*. **Supplementary Table S4.** BM28 and BM28 *ΔlysU* mutation details. **Supplementary Table S5.** High temperature growth scores of BM28-related cells with and without pOF39. **Supplementary Table S6.** Smaller BM28 and BM28 *ΔlysU* indels. **Supplementary Table S7.** PANTHER Overrepresentation Test of BM28 mutations using the Annotation Data Set GO cellular component complete. **Supplementary Table S8.** PANTHER Overrepresentation Test of BM28 *ΔlysU* mutations using the Annotation Data Set GO cellular component complete. **Supplementary Table S9.** Motility of DH10B, MG1655, BM28 and BM28 *ΔlysU*. **Supplementary Figure S1.** The 12 bp of homology between *dinB* and *mhpE*. **Supplementary Figure S2.** The O-antigen deletion in BM28 and BM28 *ΔlysU*. **Supplementary Figure S3.** BM28 and BM28 *ΔlysU* SNP mutation spectra. **Supplementary Figure S4.** Differences between BM28 and BM28 *ΔlysU*. **Supplementary Figure S5.** Growth of BM28 and BM28 *ΔlysU* on LB agar (left) and LB + Cb agar (right). **Supplementary Figure S6.** DH10B BM28 gDNA transformation plate. **Supplementary Figure S7.** PCR of BM28 gDNA using pOF39-specific primers. **Supplementary Figure S8.** Alignment of wildtype IS10R, wildtype IS10L and the IS10L/R hybrid from the BM28 Tn10. **Supplementary Figure S9.** Alignment of wildtype IS10R, wildtype IS10L and the IS10L/R hybrid from the BM28 *ΔlysU* Tn10. **Supplementary Figure S10.** Sequence logo of the IS10R target sequence, made using WebLogo (https://weblogo.berkeley.edu/logo.cgi). **Supplementary Figure S11.** Rho T96 is located on the surface of the protein and does not contact RNA polymerase nor other Rho monomers. **Supplementary Figure S12.** RpoC A595 is located on the surface of the protein and does not contact other RNA polymerase components, nor Rho. **Supplementary Figure S13.** RpoC T1135 is in close proximity to DksA, namely, DksA residue D90 (the closest distance, represented by a pink dotted line, is 7.1 Å). **Supplementary Figure S14.** RpoC T1135, DksA and a ppGpp molecule are fairly close to the active site magnesium ion of the RNA polymerase.

**Additional file 2.**

## Data Availability

All data are contained in the manuscript, the Additional Files, or submitted to GenBank. Raw short BM28 *ΔlysU* reads (SRR21641526), short BM28 reads (SRR21641525) and long BM28 reads (SRR21641524) were deposited to the SRA archive at Genbank, and final genome sequences were deposited in Genbank (JB41 CP102378.1, BM28 *ΔlysU* CP102379.1 and BM28 CP102380.1). All WGS files can be accessed from the Genbank BioProject PRJNA865726 (https://www.ncbi.nlm.nih.gov/bioproject/PRJNA865726). The pOF39 sequence (lacking the T —> C SNP) was also submitted to Genbank (OP156992.1, https://www.ncbi.nlm.nih.gov/nuccore/2310681246).

## Abbreviations

ALE: Adaptive laboratory evolution
gDNA: Genomic DNA
(p)ppGpp: ppGpp and/or pppGpp
RNAP: RNA polymerase
T_max_: Maximum growth temperature
T_min_: Minimum growth temperature
T_opt_: Optimum growth temperature
WGS: Whole-genome sequencing
