# Supplementary material for: Whole-genome sequencing analysis of two heat-evolved Escherichia coli strains
Source: BMC Genomics. 2023 Mar 27;24:154. doi: 10.1186/s12864-023-09266-9 (PMC10044804; doi:10.1186/s12864-023-09266-9)
Supplement: Supplementary file 1 — Additional file 1: Supplementary Table S1. BM28 and BM28 ΔlysU NextSeq 2000 Illumina WGS data statistics. Supplementary Table S2. BM28 Oxford Nanopore WGS data statistics. Supplementary Table S3. Large deletions in BM28 and BM28 ΔlysU. Supplementary Table S4. BM28 and BM28 ΔlysU mutation details. Supplementary Table S5. High temperature growth scores of BM28-related cells with and without pOF39. Supplementary Table S6. Smaller BM28 and BM28 ΔlysU indels. Supplementary Table S7. PANTHER Overrepresentation Test of BM28 mutations using the Annotation Data Set GO cellular component complete. Supplementary Table S8. PANTHER Overrepresentation Test of BM28 ΔlysU mutations using the Annotation Data Set GO cellular component complete. Supplementary Table S9. Motility of DH10B, MG1655, BM28 and BM28 ΔlysU. Supplementary Figure S1. The 12 bp of homology between dinB and mhpE. Supplementary Figure S2. The O-antigen deletion in BM28 and BM28 ΔlysU. Supplementary Figure S3. BM28 and BM28 ΔlysU SNP mutation spectra. Supplementary Figure S4. Differences between BM28 and BM28 ΔlysU. Supplementary Figure S5. Growth of BM28 and BM28 ΔlysU on LB agar (left) and LB + Cb agar (right). Supplementary Figure S6. DH10B BM28 gDNA transformation plate. Supplementary Figure S7. PCR of BM28 gDNA using pOF39-specific primers. Supplementary Figure S8. Alignment of wildtype IS10R, wildtype IS10L and the IS10L/R hybrid from the BM28 Tn10. Supplementary Figure S9. Alignment of wildtype IS10R, wildtype IS10L and the IS10L/R hybrid from the BM28 ΔlysU Tn10. Supplementary Figure S10. Sequence logo of the IS10R target sequence, made using WebLogo (https://weblogo.berkeley.edu/logo.cgi). Supplementary Figure S11. Rho T96 is located on the surface of the protein and does not contact RNA polymerase nor other Rho monomers. Supplementary Figure S12. RpoC A595 is located on the surface of the protein and does not contact other RNA polymerase components, nor Rho. Supplementary Figure S13. RpoC T1135 is in clos [file 12864_2023_9266_MOESM1_ESM.docx]

**SUPPLEMENTARY**

For manuscript ‘Whole-genome sequencing analysis of two heat-evolved *Escherichia coli* strains’

**Supplementary Table S1**. BM28 and BM28 *ΔlysU* NextSeq 2000 Illumina WGS data statistics.

| Strain | Q30 paired-end reads that mapped to MG1655 reference (total Q30 reads) | Mean coverage when mapped to reference genome | Standard deviation of coverage | Minimum coverage | Maximum coverage | Mean read lengths | Mean pair distance |
| --- | --- | --- | --- | --- | --- | --- | --- |
| BM28 | 5,242,394 (5,301,358) | 133.9 | 54.1 | 0 | 829 | 119.0 | 223 |
| BM28 *ΔlysU* | 3,617,932 (3,643,332) | 85.0 | 23.2 | 0 | 388 | 109.3 | 327 |

**Supplementary Table S2.** BM28 Oxford Nanopore WGS data statistics.

| Number of reads | Mean length | Standard deviation of length | Minimum length | Maximum length | Number of bases covered by reads | Mean coverage per base |
| --- | --- | --- | --- | --- | --- | --- |
| 944,944 | 2,884.2 | 3,791.9 | 1 | 98,478 | 2,725,526,891 | 587.2 |

**Supplementary Table S3.** Large deletions in BM28 and BM28 *ΔlysU*.

| Deletion name (length of deletion) | Genes affected (number of genes affected) | b numbers of affected genes | Notes |
| --- | --- | --- | --- |
| *dinB-mhpE* deletion (123 kb) | *dinB-mhpE* (134) | b0231-352 | this deletion fuses the *dinB* and *mhpE* genes in-frame |
| e14 phage excision (15 kb) | *ymfD-mcrA* (25) | b1137-59, b4692, b4693 | standard excision of e14 |
| O-antigen gene deletion (8.5 kb) | *wbbL-rfbD* (10) | b4571, b2032-40 | partially deletes the already interrupted *wbbL* and intact *rfbD* genes |

**Supplementary Table S4.** BM28 and BM28 *ΔlysU* mutation details.

|  | **BM28** | **BM28 *ΔlysU*** |
| --- | --- | --- |
| **Total changes compared to MG1655** | **233** | **244** |
| **Total changes compared to JB41** | **231** | **242** |
| Number of SNPs, indels and substitutions ≤5 bp | 208 | 220 |
| SNPs | 151 | 164 |
| Transitions | 134 | 138 |
| Transversions | 17 | 26 |
| Deletions >2 kb | 3 | 3 |
| Insertion sequence deletions and duplications | 17 | 16 |
| Indels >6 bp | 3 | 3 |
| **Average # of changes per 24 hours** | **0.092** | **0.096** |

**Supplementary Table S5.** High temperature growth scores of BM28-related cells with and without pOF39. MG1655, BM28 *ΔlysU* and BM28c, with and without pOF39, were streaked onto LB agar plates and incubated at the temperatures indicated for 48 hours. Their growth was scored using the following system: -: no colonies, +: high percent killing, ++: some percent killing, +++: growth (without percent killing) and ++++: robust growth. Growth scores that represent what we consider to be true growth (+++ and ++++) are shown in bold.

| Strain | 46.9-47.0 °C | | 47.2-47.3 °C | |
| --- | --- | --- | --- | --- |
|  | no pOF39 | with pOF39 | no pOF39 | with pOF39 |
| MG1655 | + | **++++** | + | ++ |
| BM28 *ΔlysU* | **+++** | **++++** | **+++** | **++++** |
| BM28c | **+++** | **++++** | **+++** | **++++** |

**Supplementary Table S6.** Smaller BM28 and BM28 *ΔlysU* indels.

| Indel name^a^ | Genes affected/genes surrounding mutation^b^ | b numbers of affected genes | Notes |
| --- | --- | --- | --- |
| mhpE/mhpT repeat region deletion | *-mhpE-> / -****mhpT->*** | b0352 / b0353 | deletion of some small repeat regions between these genes (202 bp deletion) |
| ybfL and ybfD deletion and fusion | *ybfL,* *ybfD* | b0705, b0706 | a partial deletion of both of these genes fuses what remains in-frame (1267 bp deletion) |
| IS1A deletion (between *flhD* and uspC) | *insA-5* | b1894 | a simple excision and rejoining of the chromosome |
| fre/fadA repeat region deletion***** | *-fre-> / <-fadA-* | b3844 / b3845 | deletion of some small repeat regions between these genes (196 bp deletion) |
| *lysU::cat***‡** | *lysU* | b4129 | *lysU* was interrupted by a *cat* gene and its promoter |
| IS5 deletion (between *ychE* and *oppA*) | *insH21* | b4711 | a simple excision and rejoining of the chromosome |

^a^Mutations only present in BM28 are indicated with an asterisk (*****) and mutations only present in BM28 *ΔlysU* are indicated with a double dagger (**‡**).

^b^Arrows surrounding genes indicate their directions and genes in bold have the insertion upstream of their start codons, meaning the insertions may affect the promoters or ribosome-binding sites of the genes.

**Supplementary Table S7.** PANTHER Overrepresentation Test of BM28 mutations using the Annotation Data Set GO cellular component complete. Genes with intragenic mutations and genes surrounding intergenic changes were used, whereas genes that were entirely deleted were excluded from the analysis.

| GO cellular component complete | Escherichia coli - REFLIST (4392) | upload_1 (239) | upload_1 (expected) | upload_1 (over/under) | upload_1 (fold Enrichment) | upload_1 (raw P-value) | upload_1 (FDR) |
| --- | --- | --- | --- | --- | --- | --- | --- |
| membrane (GO:0016020) | 1455 | 112 | 79.18 | + | 1.41 | 2.27E-05 | 6.21E-03 |
| cellular anatomical entity (GO:0110165) | 2908 | 186 | 158.24 | + | 1.18 | 1.73E-04 | 1.19E-02 |
| Unclassified (UNCLASSIFIED) | 1423 | 49 | 77.44 | - | 0.63 | 8.17E-05 | 7.46E-03 |

**Supplementary Table S8.** PANTHER Overrepresentation Test of BM28 *ΔlysU* mutations using the Annotation Data Set GO cellular component complete. Genes with intragenic mutations and genes surrounding intergenic changes were used, whereas genes that were entirely deleted were excluded from the analysis.

| GO cellular component complete | Escherichia coli - REFLIST (4392) | upload_1 (255) | upload_1 (expected) | upload_1 (over/under) | upload_1 (fold Enrichment) | upload_1 (raw P-value) | upload_1 (FDR) |
| --- | --- | --- | --- | --- | --- | --- | --- |
| membrane (GO:0016020) | 1455 | 120 | 84.48 | + | 1.42 | 8.66E-06 | 2.37E-03 |
| integral component of membrane (GO:0016021) | 1111 | 89 | 64.5 | + | 1.38 | 9.05E-04 | 4.96E-02 |
| cellular anatomical entity (GO:0110165) | 2908 | 196 | 168.84 | + | 1.16 | 3.62E-04 | 2.48E-02 |
| Unclassified (UNCLASSIFIED) | 1423 | 53 | 82.62 | - | 0.64 | 7.50E-05 | 6.85E-03 |

**Supplementary Table S9.** Motility of DH10B, MG1655, BM28 and BM28 *ΔlysU*. The motility of the strains was determined using a soft agar stab assay, in duplicate.

| Strain | Motile? |
| --- | --- |
| DH10B | yes |
| MG1655 | yes |
| BM28 | no |
| BM28 *ΔlysU* | no |

**
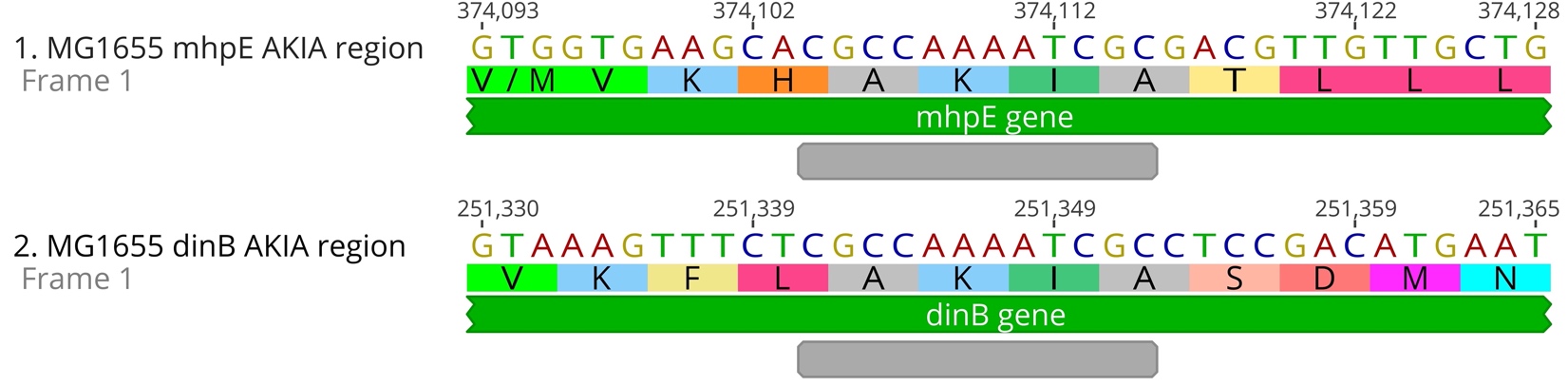
**

**Supplementary Figure S1.** The 12 bp of homology between *dinB* and *mhpE*. Grey rectangles mark the 12 bp of identical DNA sequence between the two genes. The amino acid sequence of these regions are also shown, showing that both genes encode the amino acid sequence ‘AKIA’ in this region.

**Supplementary Figure S2.** The O-antigen deletion in BM28 and BM28 *ΔlysU*. **A.** Alignment of the O-antigen region in MG1655 (top) and BM28 (bottom). The two parallel horizontal black lines in between the *insH* element and *rfbD* in the BM28 genome represent a gap in the alignment. **B.** The site of the O-antigen gene deletion in BM28 and BM28 *ΔlysU*. The exact site of the deletion and reclosing of the chromosome is between the 5’ end of the mobile element IS5 (shown in purple) and a base partway through the *rfbD* gene (genes shown in green).

**Supplementary Figure S3.** BM28 and BM28 *ΔlysU* SNP mutation spectra.

**Supplementary Figure S4.** Differences between BM28 and BM28 *ΔlysU*. Differences larger than 6 bp are shown in the outer ring and labeled, including *lysU::cat*, the *fre/fadA* deletion and the *fliZ* IS10R interruption. The middle ring shows small BM28 *ΔlysU* specific changes and the innermost ring shows small BM28 specific changes. Blue arrowheads represent BM28 specific changes and yellow arrowheads represent BM28 *ΔlysU* specific changes.


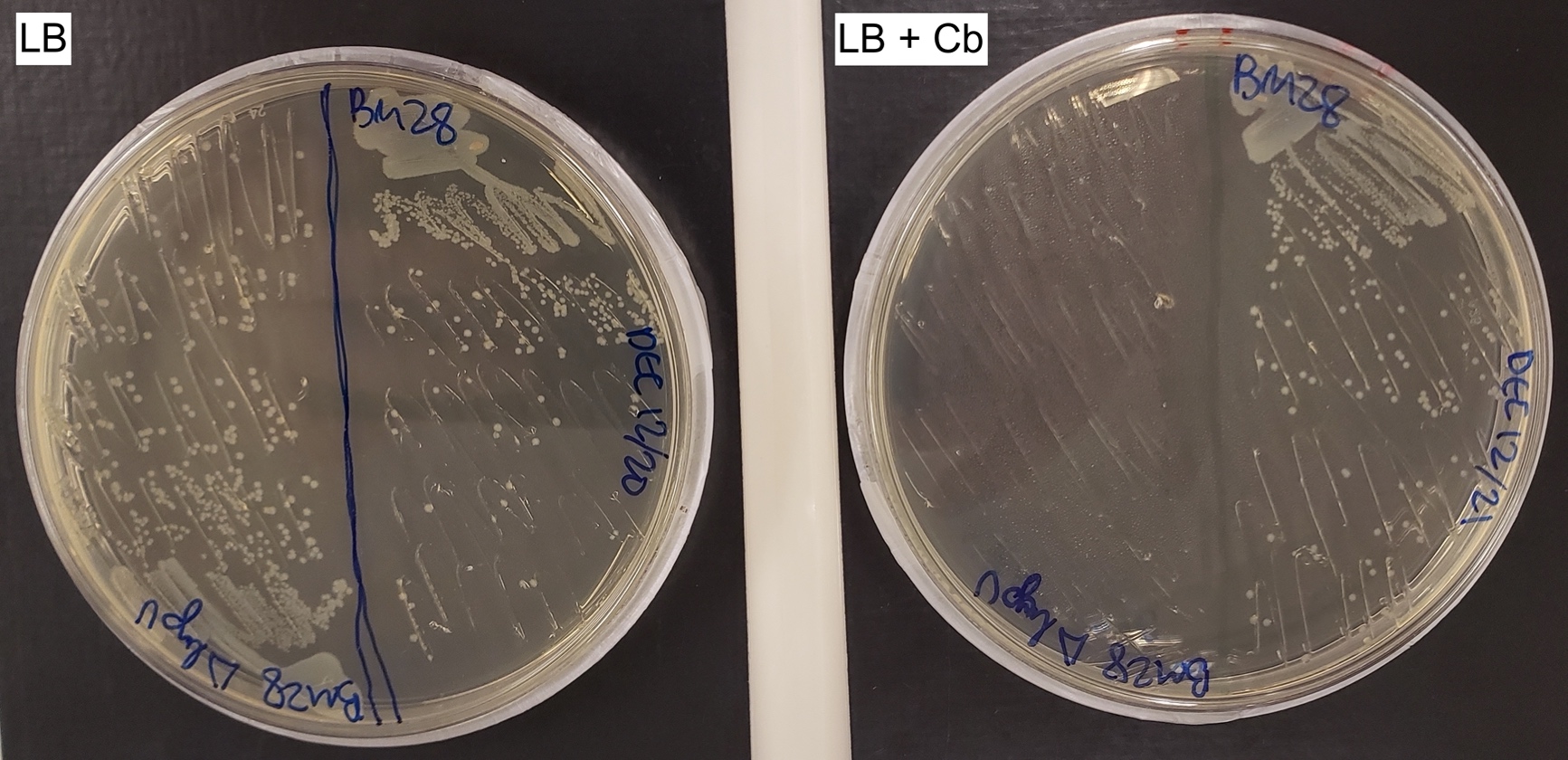


**Supplementary Figure S5.** Growth of BM28 and BM28 *ΔlysU* on LB agar (left) and LB + Cb agar (right). Cells were streaked from glycerol stocks and the plates were incubated overnight at 37 °C. No colonies arose for BM28 *ΔlysU* on LB + Cb plates. The cells were streaked out on the same day, the left plate’s date should read Dec 12/21 but instead says Dec 12/20 in error.


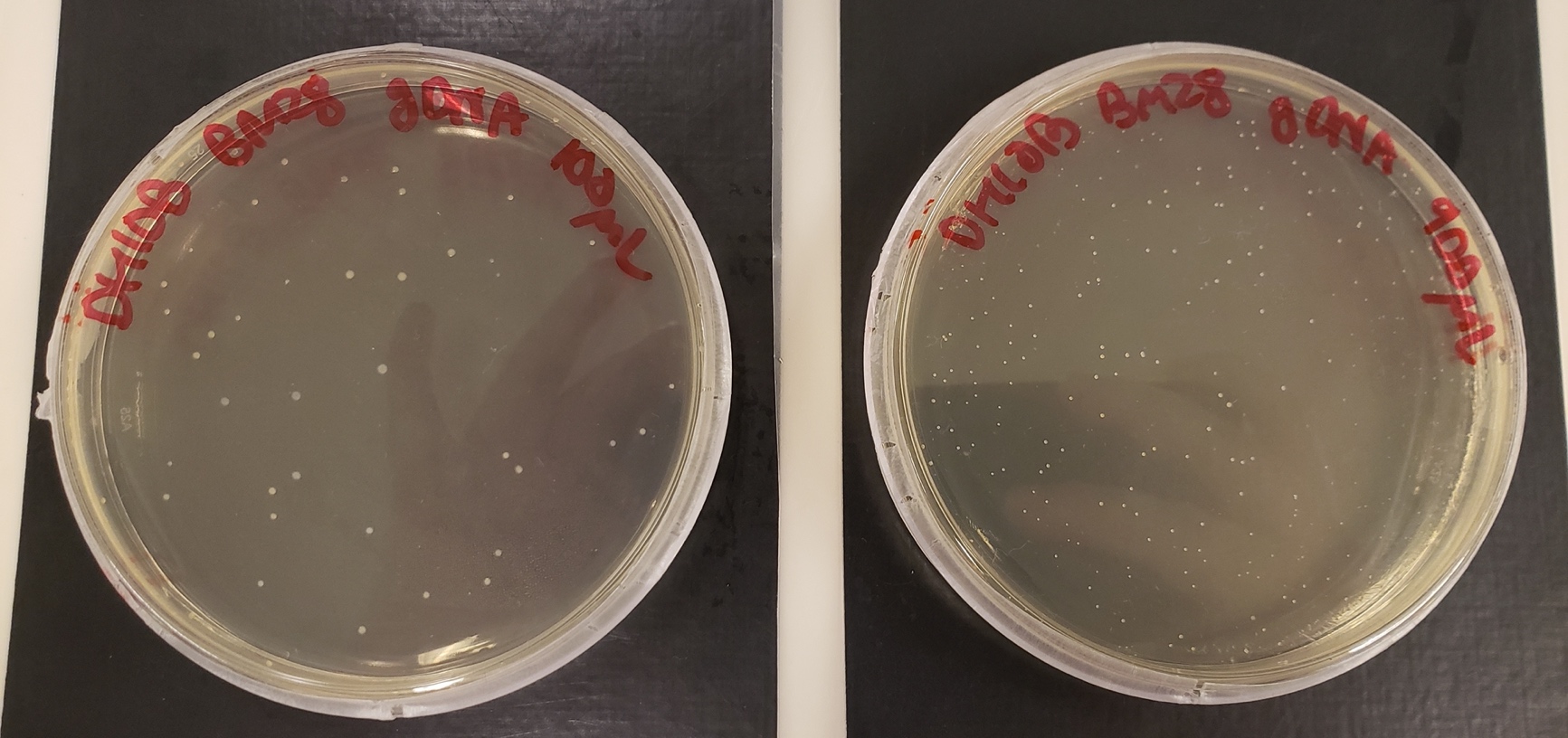


**Supplementary Figure S6.** DH10B BM28 gDNA transformation plate. Chemically competent DH10B was transformed with BM28 gDNA and 0.1 mL of the 1 mL transformation mixture was spread plated on LB + Cb plates and incubated at 37 °C overnight.


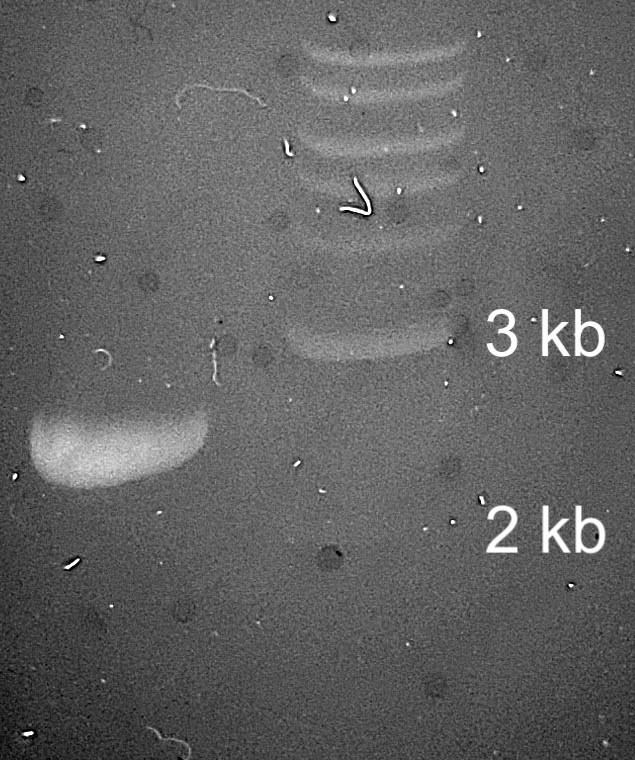


**Supplementary Figure S7.** PCR of BM28 gDNA using pOF39-specific primers. The expected length of the pOF39 PCR product is 2,367 bp.


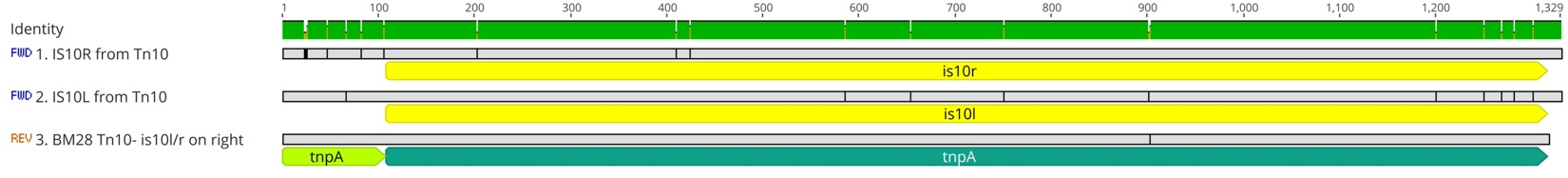


**Supplementary Figure S8**. Alignment of wildtype IS10R, wildtype IS10L and the IS10L/R hybrid from the BM28 Tn10. An identity map is shown along the top in green. Black lines indicate a different base in that position compared to the most common base seen in the three sequences. This IS10L/R hybrid is essentially IS10L for the promoter and first third of the transposase ORF, and then essentially IS10R for the last two thirds of the transposase ORF.


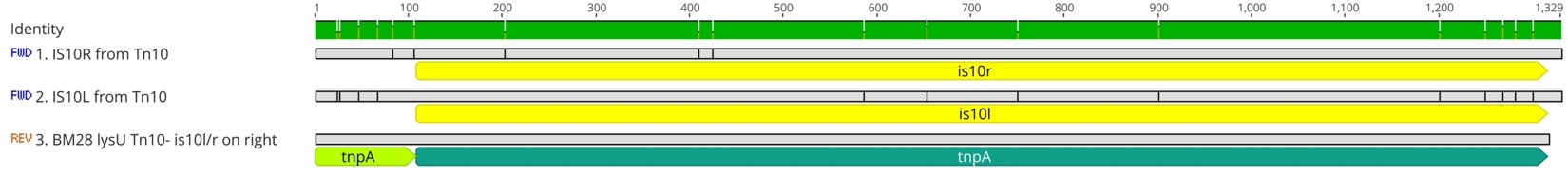


**Supplementary Figure S9**. Alignment of wildtype IS10R, wildtype IS10L and the IS10L/R hybrid from the BM28 *ΔlysU* Tn10. An identity map is shown along the top in green. Black lines indicate a different base in that position compared to the most common base seen in the three sequences. This IS10L/R hybrid is essentially IS10R for the first two thirds of the promoter, IS10L for the last third of the promoter and first third of the transposase ORF, and then essentially IS10R for the last two thirds of the transposase ORF.

**Supplementary Figure S10.** Sequence logo of the IS10R target sequence, made using WebLogo (https://weblogo.berkeley.edu/logo.cgi). For the 15 IS10R copies in BM28 not part of the Tn10 transposon, the 9 bp target sequences that IS10R inserted into were used to make a sequence logo.


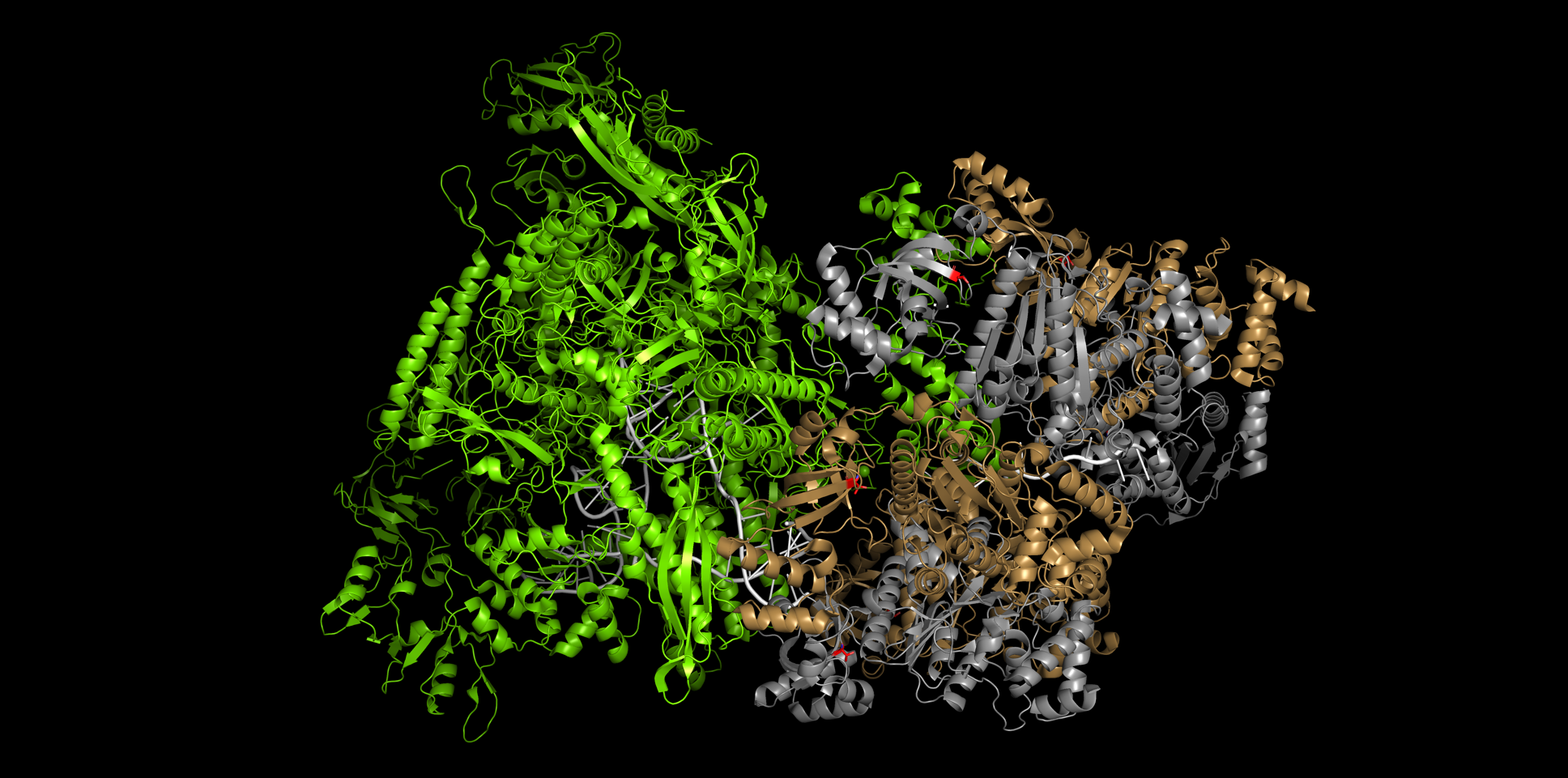


**Supplementary Figure S11.** Rho T96 is located on the surface of the protein and does not contact RNA polymerase nor other Rho monomers. The RNA polymerase is shown in chartreuse, the Rho subunits are shown in alternating grey and brown and Rho T96 is shown in red in each hexamer subunit (PDB 6XAS). Generated using PyMOL 2.5.

**
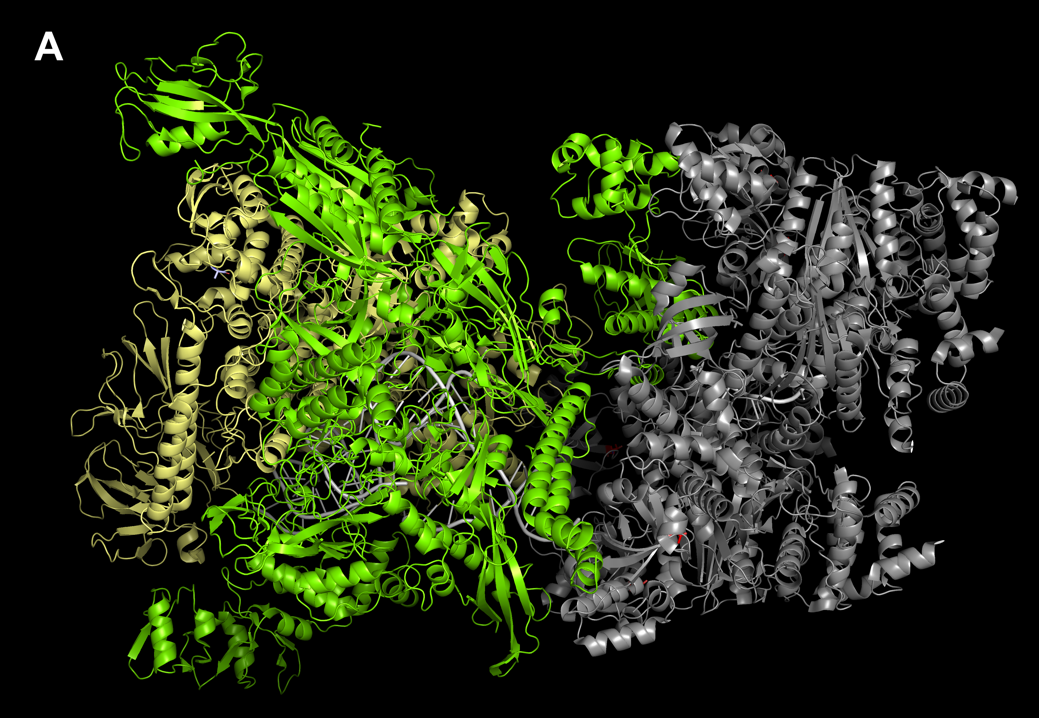
**

**
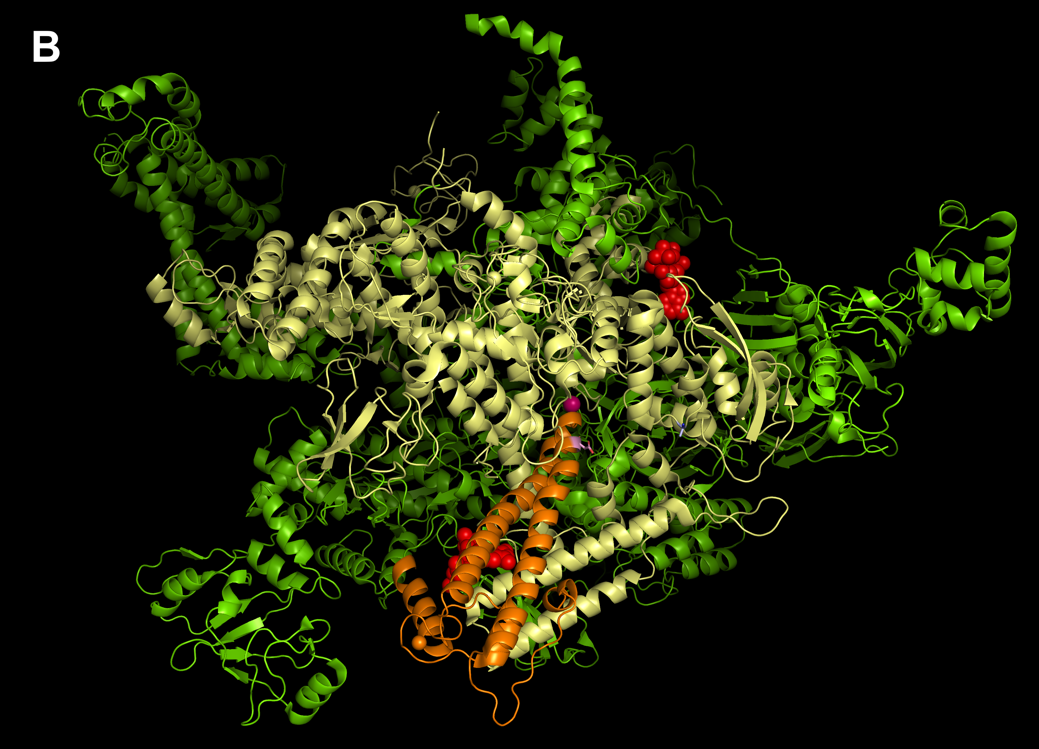
**

**
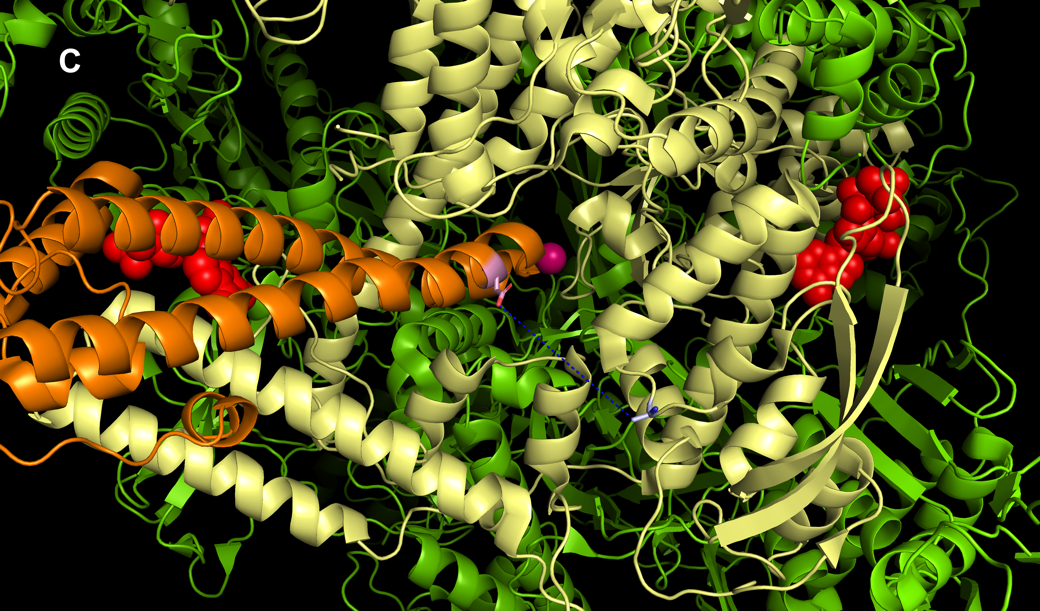
**

**Supplementary Figure S12. A.** RpoC A595 is located on the surface of the protein and does not contact other RNA polymerase components, nor Rho. RpoC is shown in light yellow, the rest of the RNA polymerase is shown in chartreuse, the Rho hexamer is shown in grey, Rho T96 is shown in red for all six subunits and RpoC A595 is shown in light blue near the top left of the figure (PDB 6XAS). **B and C.** RpoC is shown in light yellow, the rest of the RNA polymerase is shown in chartreuse, DksA is shown in orange, the active site magnesium ion is shown in pink, the ppGpp molecules are shown in red, RpoC A595 is shown in light blue and DksA D64 is shown in light purple (PDB 5VSW). **B.** RpoC A595 also does not contact DksA (PDB 5VSW). **C.** RpoC A595 and DksA D64 are fairly close to each other (the closest distance, represented by a blue dotted line, is 16.6 Å). Generated using PyMOL 2.5.


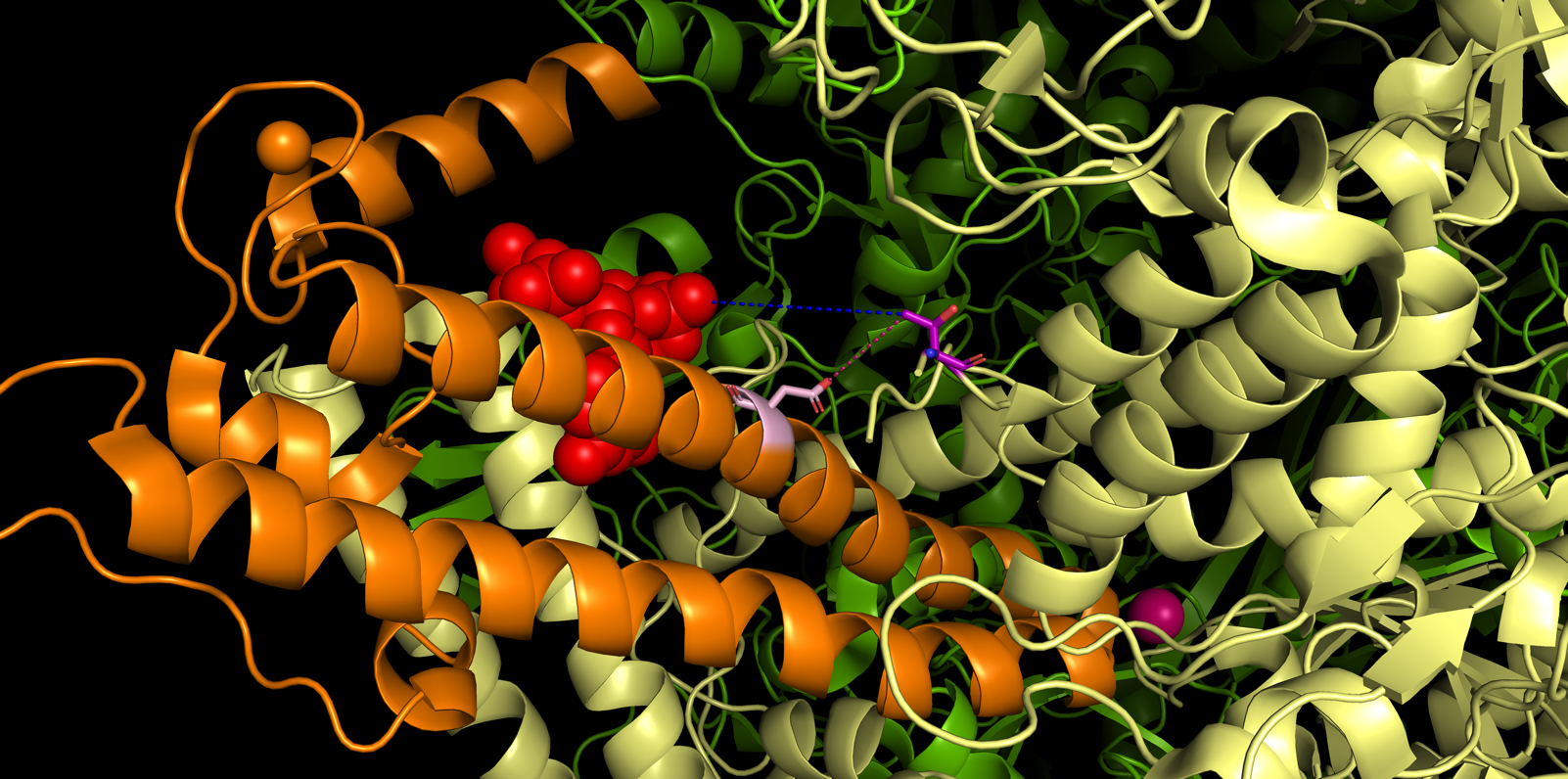


**Supplementary Figure S13.** RpoC T1135 is in close proximity to DksA, namely, DksA residue D90 (the closest distance, represented by a pink dotted line, is 7.1 Å). T1135 is also fairly close to a RNA polymerase ppGpp binding site (the closest distance, represented by a blue dotted line, is 13.4 Å). RpoC is shown in light yellow, the rest of the RNA polymerase is shown in chartreuse, DksA is shown in orange, the active site magnesium ion is shown in pink, the ppGpp molecules are shown in red, RpoC T1135 is shown in purple and DksA D90 is shown in light pink (PDB 5VSW). Generated using PyMOL 2.5.


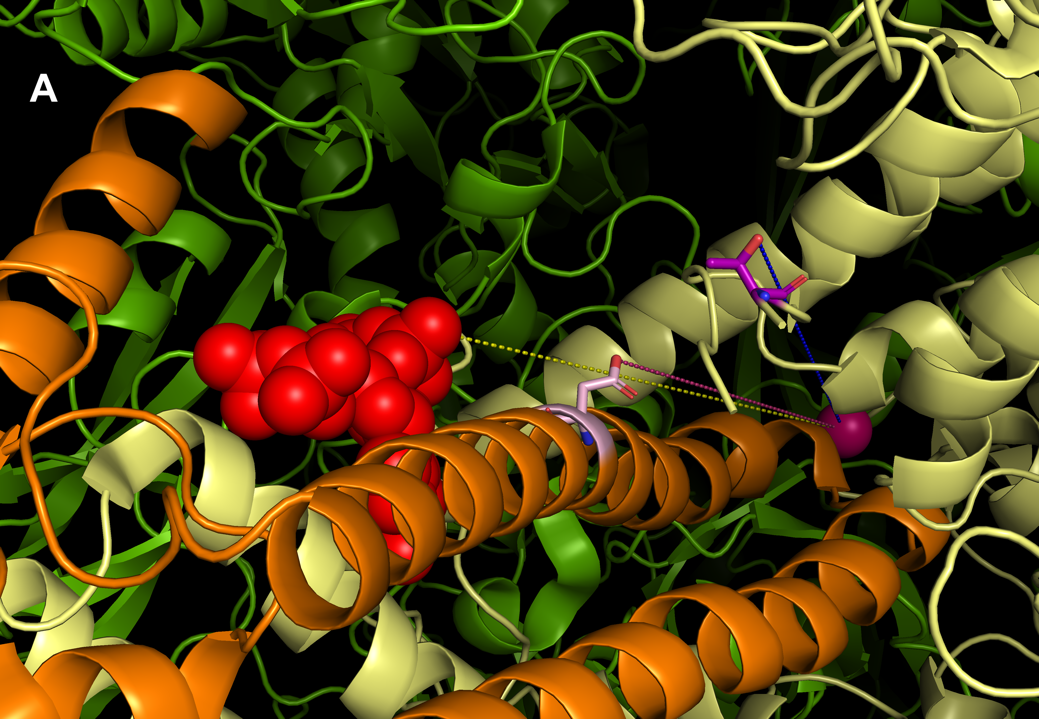


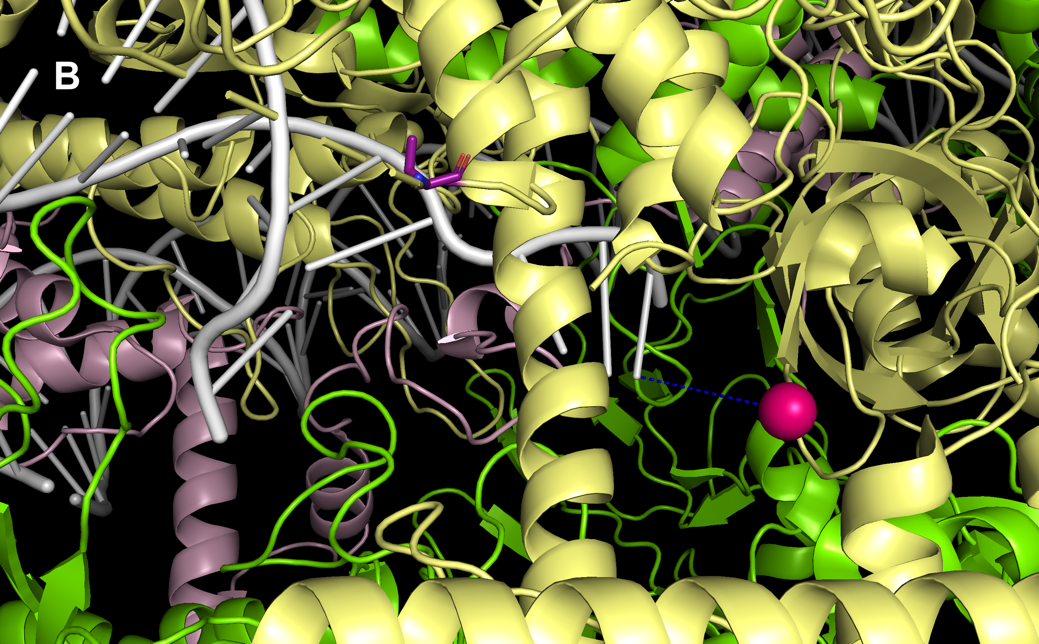


**Supplementary Figure S14. A.** RpoC T1135, DksA and a ppGpp molecule are fairly close to the active site magnesium ion of the RNA polymerase. RpoC is shown in light yellow, the rest of the RNA polymerase is shown in chartreuse, DksA is shown in orange, the active site magnesium ion is shown in pink, the ppGpp molecules are shown in red, RpoC T1135 is shown in purple and DksA D90 is shown in light pink (PDB 5VSW). The distance between the magnesium ion and RpoC T1135 is 33.5 Å (represented by a blue dotted line), the distance between the magnesium ion and DksA D90 is 31.5 Å (represented by a pink dotted line) and the distance between the magnesium ion and the ppGpp molecule is 37.6 Å (represented by a yellow dotted line). **B.** The active site magnesium ion and the DNA in open promoter complexes are close to each other (the closest distance, represented by a blue dotted line, is 11.3 Å). RpoC is shown in light yellow, the rest of the core RNA polymerase is shown in chartreuse, the RpoD sigma factor is shown in light pink, the DNA is shown in white, the active site magnesium ion is shown in pink and RpoC T1135 is shown in purple (PDB 6OUL). Generated using PyMOL 2.5.
